# Voltage sensor gating charge interactions bimodally regulate voltage-dependence and kinetics of calcium channel activation

**DOI:** 10.1085/jgp.202513769

**Published:** 2025-08-04

**Authors:** Martin C. Heiss, Monica L. Fernandeź-Quintero, Marta Campiglio, Yousra El Ghaleb, Simone Pelizzari, Johannes R. Loeffler, Klaus R. Liedl, Petronel Tuluc, Bernhard E. Flucher

**Affiliations:** 1 Institute of Physiology, Department of Physiology and Medical Biophysics, Medical University Innsbruck, 6020 Innsbruck, Austria; 2 Institute of General, Inorganic and Theoretical Chemistry, University of Innsbruck, Innsbruck, Austria; 3 Department of Pharmacology and Toxicology, Center for Molecular Biosciences Innsbruck, University of Innsbruck, 6020 Innsbruck, Austria

## Abstract

Voltage-sensing domains (VSDs) are highly conserved protein modules that regulate the activation of voltage-gated ion channels. In response to membrane depolarization positive gating charges in the S4 helix of VSDs move across the membrane electric field, which is focused at the hydrophobic constriction site in the center of the VSD. This conformational change is translated into opening of the channel gate. Transient interactions of the gating charges with negatively charged countercharges in the adjacent helices are critical for catalyzing this state transition and for determining its voltage-dependence and kinetics. However, the mechanism by which the sequential interactions between the multiple gating- and countercharges regulate these properties remains poorly understood. Here we analyzed the state-transitions of the first VSD of Ca_V_1.1 using MD simulation of the channel exposed to an electric field and site directed mutagenesis of gating- and countercharges to investigate the role of their interactions in determining the gating properties of Ca_V_1.1. Alanine-substitutions of gating charges differentially altered the kinetics or voltage-dependence of activation, depending on whether they pass the hydrophobic constriction site (HCS) (R2, R3) or not (K0, R1, R4). Alanine-substitutions of countercharges differentially altered kinetics and voltage-dependence, depending on whether they facilitate the transfer of gating charges across the HCS (E100, D126), and whether they stabilize the activated (E87, E90, E140) or the resting state (E100, D126). Thus, our results reveal basic mechanistic principles by which variable interactions between gating charges and countercharges regulate the gating properties of voltage-gated calcium channels.

## Introduction

The coordinated flow of ionic currents during action potentials and the activation of cell functions by electrical signals depends on the ability of ion channels to sense changes of the membrane potential. A universal structural module, called the voltage-sensing domain (VSD), has evolved, which is utilized for this purpose in a wide range of voltage-gated ion channels as well as in voltage-sensing phosphatases (Catterall et al., 2017). Its highly conserved molecular fold consists of four membrane-spanning α-helices (segments S1, S2, S3 and S4). The S4 helix carries four to six positively charged amino acids in every third position and thus functions as the voltage sensor proper. Its principle mode of action is described by the sliding helix model (Catterall, 1986). According to this widely accepted model, the membrane potential at rest pulls the positively charged S4 helix down toward the cytoplasmic side. Upon depolarization this pull is released and the S4 helix moves up relative to helices S1-S3. The associated conformational change is translated into gating of the channel pore and consequently into the activation and deactivation of ionic currents.

**Figure 1:**
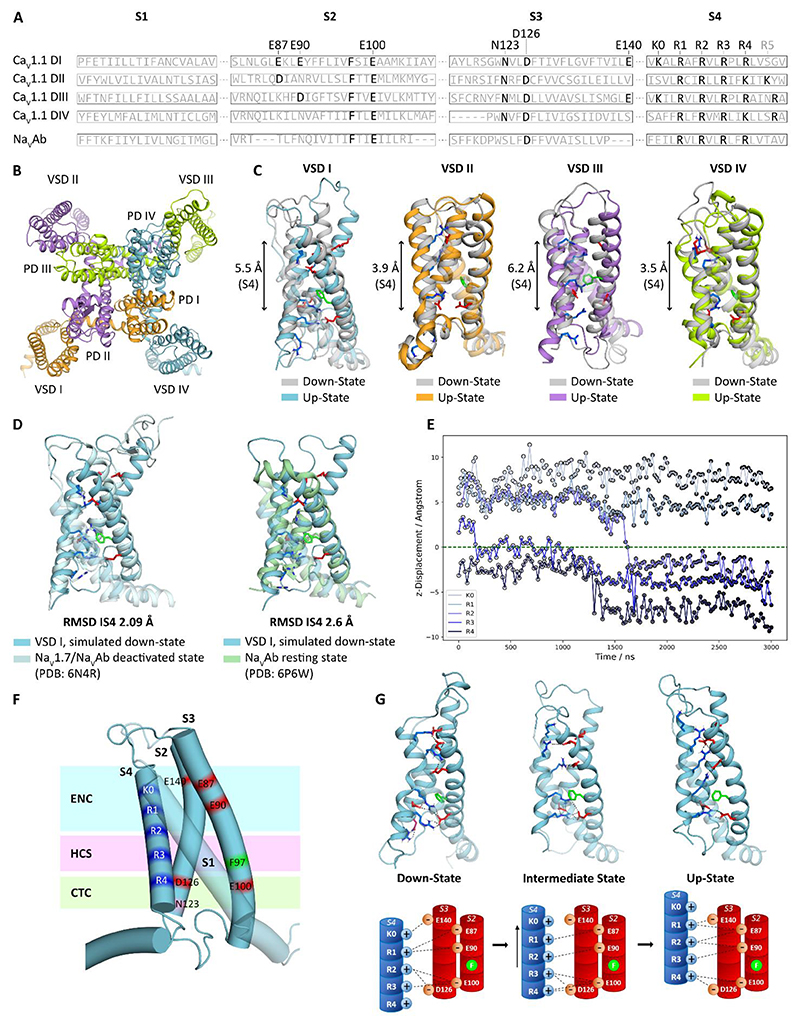
Structure of Ca_V_1.1 and molecular dynamics (MD) simulation of VSD I moving from the activated/up-state to the resting/down-state in response to an applied electric field. The CaV1.1 structure in the up-state was modeled from the cryo-EM structure PDB: 5GJW (Wu et al., 2016). Intermediate and down-states were obtained from MD simulation of Ca_V_1.1 in a membrane environment exposed to an electric field. **A**: Sequence alignment of the four transmembrane helices (S1, S2, S3, S4) of the four VSDs (I – IV) of Ca_V_1.1. S4 gating charges and countercharges in S2 and S3 are indicated (residue numbers above for VSD I). The residues conserved in the sequence of Na_V_Ab are shown for comparison. **B**: Top view of the structure of Ca_V_1.1 in the up-state. **C**: Structure overlay of the four VSDs in the up- and deepest resting/down-states obtained in ˜3 µs MD simulations. **D**: Overlay of the simulated VSD I down-state structure and the experimental structures of a Na_V_Ab/Na_V_1.7 chimera in the deactivated state (PDB: 6N4R) and of Na_V_Ab in a disulfide-locked resting state (PDB: 6P6W). **E**: Downward displacement of the IS4 gating charges relative to the position of the HCS F97 (dashed line) over the simulation time. **F**: Schematic structure of VSD I depicting the relevant structural features: ENC, extracellular negative cluster; HCS, hydrophobic constriction site with its central phenylalanine (F97); CTC, charge transfer center; S1-S4, transmembrane helices; K0 and R1-4 (blue), gating charges; red, countercharges. **G**: Structure of VSD I in the activated/up-, intermediate and resting/down-states, and schematic models (below) highlighting interactions between gating charges and countercharges.

As the S4 helix moves up and down in the VSD, its positively charged amino acids cross the membrane electric field, which is focused at the hydrophobic constriction site (HCS) (Ahern and Horn, 2005). This transition of the gating charges can be measured as gating currents and the effective charge moved is reflected by the voltage-sensitivity of charge movement or channel gating (Bezanilla, 2018). However, the transition of charged amino acids across the HCS is energetically highly unfavorably (Chanda and Bezanilla, 2008). Therefore, a central proposition of the sliding helix model is that transient formation of ion-pairs between the positive gating charges and negative countercharges on both sides of the HCS facilitate the charge transition across the HCS in response to changes of the membrane electric field (Wisedchaisri et al., 2021; Groome and Bayless-Edwards, 2020). The charge transfer center (CTC)—a highly conserved structure consisting of two negative countercharges in S2 and S3 just below the conserved phenylalanine specifying the HCS—appears to be of central importance for this action (Tao et al., 2010). Further ion-pair partners of gating charges have been identified extracellularly of the HCS and were subsumed under the terms extracellular negatively charged region or cluster (ENC) (Fig. 1 A, F). Although, the number and positions of these outer countercharges differ considerably among the studied ion channels.

In voltage-gated calcium channels (Ca_V_), we identified functionally important countercharges on the extracellular side of the HCS (Tuluc et al., 2016b; Fernández-Quintero et al., 2021; El Ghaleb et al., 2021; Costé de Bagneaux et al., 2018). These extracellular countercharges are located in the S2 and S3 helices of the respective VSD as well as in the S5 helix of the adjacent pore domain of the next repeat. As opposed to the highly conserved countercharges of the CTC, the countercharges of the ENC vary greatly in number and position between the ten members of the Ca_V_ family, as well as between the four VSDs of individual channels. In fact, conservation across the Ca_V_ genes is greater than that between the channel´s four VSDs (Fig. 1A, C). Molecular dynamics simulations suggest that the outer gating charges form transient interactions with these countercharges during the upward motion of the S4 helix and finally stabilize the VSD in the activated state. Mutagenesis experiments indicate that the ENC interactions are important determinants of the distinct gating properties of the different Ca_V_ channels. Specifically, in Ca_V_1.1 transient interactions of gating charges and a single countercharge in VSD I account for its characteristically slow activation kinetics, and multiple such interactions in VSD IV regulate the voltage-dependence of current activation by alternative splicing (Tuluc et al., 2016b; Fernández-Quintero et al., 2021). Together these findings suggest that the highly conserved countercharges of the CTC embody the common structural theme of VSDs, whereas the variable countercharges located extracellularly of the HCS represent the variations to this theme, which allow for the wide range of gating properties observed among the members of the Ca_V_ channel family. However, the molecular principles of how the ionic interactions of the conserved and variable countercharges interact to facilitate the VSD state transitions and determine the specific gating properties are still elusive.

Here we address the question as to how the transient formation of ion-pairs between the gating charges and the countercharges of the two sites (CTC and ENC) cooperate in the voltage-sensing process. In that we ask, whether general mechanistic principles can be deduced, which govern the voltage-sensing process and regulate the kinetics and voltage-dependence of the VSD state transitions and consequently of channel gating. For our study of gating charge – countercharge interactions we chose the first VSD of Ca_V_1.1e. Previously, an allosteric model based on a voltage-clamp fluorometry study in the laboratory of Riccardo Olcese showed that VSD I is the chief player in Ca_V_1.1a channel activation, whereas the other VSDs contribute only minimally (Savalli et al., 2021). However, in the embryonic splice variant Ca_V_1.1e excision of exon 29 from VSD IV substantially increased the current amplitude and voltage-dependence of activation, indicating a contribution of VSD IV to channel gating (Tuluc et al., 2009, 2016a). Nevertheless, our previous studies demonstrated that the action of VSD I regulates both the activation kinetics and voltage-dependence of current activation (Fernández-Quintero et al., 2021). Thus, analysis of current properties can be utilized as proxy of Ca_V_1.1e VSD I function.

In line with recent cryo-EM structures (Wu et al., 2016; Wisedchaisri et al., 2019), our molecular modeling shows that only the two middle gating charges (R2, R3) of VSD I fully pass the HCS, while the outer- and innermost gating charges remain on the extracellular and intracellular side, respectively. Accordingly, our mutagenesis experiments demonstrate a differential behavior of those gating interactions involved in charge transfer across the membrane electric field and those involved in stabilizing the activated and/or resting states. While disruption of ion-pairs involved in charge transfer across the HCS primarily affected the speed of activation, the balance between intracellular and extracellular ion-pairs stabilizing the activated and the resting states determined the voltage-dependence of activation. These findings extend the sliding helix model in that they reveal molecular mechanisms by which the intricate interactions of gating charges with conserved and variable countercharges tune the VSD state transitions in response to depolarization and thus determine the characteristic gating properties of Ca_V_ channels.

## Materials and methods

### Plasmids

Cloning procedures for GFP-Ca_V_1.1e (Genbank NM_001101720) and GFP-Ca_V_1.1e-R174A were previously described (Pelizzari et al., 2024). Sanger sequencing (Eurofins Genomics) verified the integrity of the newly generated plasmid.

GFP-Ca_V_1.1e-K162A, -R165A, -R168A, -R171A, -E100A, -D126A, -N123A, -E140A, -N123A/D126A

To generate the desired constructs, each mutation was introduced into GFP-Ca_V_1.1e by splicing by overlap extension (SOE) PCR. First, the cDNA sequence of rabbit Ca_V_1.1 (nt 1–1006) was amplified in separate PCR reactions using GFP-Ca_V_1.1e as the template, employing overlapping primers that introduced the mutations. The two resulting PCR products were then used as templates for a PCR reaction with flanking primers to connect the nucleotide sequences. They were then combined in a subsequent reaction using flanking primers to connect the nucleotide sequences. The final fragment was digested with SalI and EcoRI and ligated into the corresponding sites of GFP-Ca_V_1.1e. The flanking primers used for all construct were: SalI-F: 5´- cgaaaagagagaccacat- 3´ and EcoRI-R: 5´- cgtgatccagctcatgta- 3.

The primers introducing the mutations were:

K162A, fw 5´- cctggacgtcgcggccctgaaggccttccgtgtgc -3´,

rev 5´- ccttcagggccgcgacgtccaggccggctcctttgc - 3´.

R165A, fw 5´- tcaaggccctggcggccttccgtgtgctcagacccc -3´,

rev 5´- acggaaggccgccagggccttgacgtccaggccggc - 3´.

R168A, fw 5´- tgaaggccttcGCtgtgctcagacccctccggctgg -3´,

rev 5´- tctgagcacaGCgaaggccttcagggccttgacgtc - 3´.

R171A, fw 5´- cttccgtgtgctcgcacccctccggctggtgtcggg -3´,

rev 5´- ccggaggggtgcgagcacacggaaggccttcagggc - 3´.

E100A, fw 5´- ttctccatcgctgcagccatgaagatcatcgcctacgg -3´,

rev 5´- ttcatggctgcagcgatggagaagacggtgaggaagaa - 3´.

D126A, fw 5´- tggaacgtgctggccttcatcatcgtcttcctgggg -3´,

rev 5´- gatgatgaaggccagcacgttccagccgctgcgcag - 3´.

N123A, fw 5´- ggccgtgctggccttcatcatcgtcttcctgggggt -3´,

rev 5´- acgatgatgaaggccagcacggcccagccgctgcgc - 3´.

E140A, fw 5´- acggcgattctggcacaggtcaacgtcatccagagc -3´,

rev 5´- gttgacctgtgccagaatcgccgtgaagacccccag - 3´.

N123A/D126A, fw 5´- gcagcggctgggccgtgctggccttcatcatcgtc -3´,

rev 5´-ggccagcacggcccagccgctgcgcaggtaggcgt - 3´.

GFP-Ca_V_1.1e-E100A/N123A/D126A, -E100A/D126A, -E100A/N123A

To generate these constructs, we employed the same cloning strategy, using GFP-Ca_V_1.1e-E100A as the template for the PCR reactions. For GFP-Ca_V_1.1e-E100A/N123A/D126A we used the primers introducing the N123A/D126A mutations, for GFP-Ca_V_1.1e-E100A/D126A we used the primers introducing the D126A mutation, and for GFP-Ca_V_1.1e-E100A/N123A we used the primers introducing the N123A mutation.

### Cell culture and transfection

Dysgenic (α1s-null) myoblasts (GLT cell line) were cultured in growth medium consisting of Dulbecco’s Modified Eagle Medium (DMEM) with 1 g/L glucose, supplemented with 10% fetal calf serum and 10% heat-inactivated horse serum (HS), in culture flasks maintained at 37 °C in a humidified incubator with 10% CO_2_. The cells were then seeded into 35 mm plastic dishes for electrophysiology experiments or onto gelatin and carbon-coated plastic dishes for immunofluorescence analysis. On the second day, and every other day thereafter, the medium was replaced with fusion medium (DMEM supplemented with 2% HS). On the fourth day, the cells were transiently transfected with a specific Ca_V_1.1 construct using FuGeneHD transfection reagent (Promega) following the manufacturer’s protocol. Electrophysiology experiments were conducted on days 7 and 8 after plating, while immunofluorescence staining was carried out on days 9 and 10.

### Immunostaining and Quantification

Paraformaldehyde-fixed cultures were immunolabeled with the polyclonal rabbit anti-GFP antibody (serum, 1:10,000; A6455 Thermofisher Scientific) and the monoclonal mouse anti-RyR (34 C, 1:200; MA3-925 Thermofisher Scientific) and subsequently fluorescently labeled with Alexa-488 and Alexa-594, respectively, as previously described (Campiglio et al., 2013). 14-bit images were recorded with a cooled CCD camera (SPOT) and Metaview image processing software. Image composites were arranged in Affinity Designer and Photo (version 2.5.3, Serif (Europe) Ltd.) and non-linear adjustments were performed to correct black level and contrast for the graphical representation. Clusters of GFP-Ca_V_1.1 and RyR were quantified using Image J software (NIH), as previously described (Nakada et al., 2018). Briefly, myotubes were selected by a ROI tool and converted to binary images using the intermodes threshold, so that only clusters are included. Using the Analyze Particle function, the numbers of particles larger than 0.2 - 10 μm^2^ in the binary image were counted as clusters. The numbers of clusters per 100 μm^2^ were calculated and are represented in the graphs. To quantify the GFP-Ca_V_1.1/RyR colocalization, the thresholded image was used as a mask on the myotube image to include only the clusters and Pearson´ s coefficients for colocalization were calculated by JaCoP, as previously described (Tuinte et al., 2022). For each condition, the number of clusters of 15 myotubes from at least three separate experiments was counted. Graphs and statistical analysis (One-way ANOVA multiple comparison) were performed using GraphPad Prism 10 software package.

### Molecular dynamics simulations

As described in Pelizzari et al., (Pelizzari et al., 2024) we predicted the structure of the human WT Ca_V_1.1 α_1_ subunit by making a homology model based on the cryo-EM structure of the rabbit Ca_V_1.1 α_1_ subunit with the VSDs in the up-state and the pore closed (Wu et al., 2016). Homology modeling was performed using Rosetta and MOE (Molecular Operating Environment, version 2020, Molecular Computing Group Inc, Montreal, Canada, (Canutescu and Dunbrack, 2003; Leman et al., 2020; Wang et al., 2007). The C-terminal and N-terminal parts of each domain were capped with acetylamide and *N*-methylamide to avoid perturbations by free charged functional groups. The structure was aligned in the membrane using the PPM server (Lomize et al., 2012) and inserted into a plasma membrane consisting of POPC (1-palmitoyl2-oleoyl-sn-glycero-3-phosphocholine) and cholesterol in a 3:1 ratio, using the CHARMM-GUI Membrane Builder (Jo et al., 2009). Water molecules and 0.15 M CaCl_2_ were included in the simulation box. For calcium the standard parameters for calcium-ions were replaced with the multi-site calcium model of Zhang et al. (Zhang et al., 2020). This multi-site model has been used to simulate calcium permeation in a number of channels, including type-1 ryanodine receptor, AMPA receptors, the E protein of SARS-CoV-2, and TRPV channels (Zhang et al., 2020; Schackert et al., 2023; Ives et al., 2023; Liu and Zukin, 2007; Antonides et al., 2022). We performed simulations of the WT Ca_V_1.1 α_1_ and E100A/D126A mutant with an external electrical field (~3 µs of simulation time).

Simulations of the WT Ca_V_1.1 α_1_ subunit and the variants were performed using GROMACS 2020.2 (Lindahl, 2020; Abraham et al., 2015) with the CHARMM36m force field for the protein, lipids and ions (Huang et al., 2017). The TIP3P water model was used to model solvent molecules (Jorgensen et al., 1983). The system was minimized and equilibrated using the suggested equilibration input scripts from CHARMM-GUI (Lee et al., 2016), i.e., the system was equilibrated using the NPT ensemble for a total time of 2 ns with force constraints on the system components being gradually released over six equilibration steps. The systems were further equilibrated by performing a 10 ns simulation with no electric field applied. To maintain the temperature at T = 310 K we used the Nosé-Hoover thermostat with the inverse friction constant set to 1.0 ps (Evans and Holian, 1985), and the pressure was maintained semi-isotropically at 1 bar using the Parrinello-Rahman barostat with the period of pressure fluctuations at equilibrium set to 5.0 ps and the compressibility set to 4.5e-5 bar^-1^ (Parrinello and Rahman, 1981). Periodic boundary conditions were used throughout the simulations. Long-range electrostatic interactions were modelled using the particle-mesh Ewald method (Darden et al., 1993) with a cut-off of 12 Å. Bond lengths involving bonds with hydrogen atoms were constrained using the LINCS algorithm (Hess, 2008). The simulations were performed using a time step of 2 fs and an applied electric field to produce membrane voltage (Aksimentiev and Schulten, 2005). An electric potential of 600 mV was applied respectively. Such unphysiologically high potentials are required to observe the VSD state transitions, which usually occur at the ms time scale, within the few µs simulation time. All applied field simulations were ~3 µs long. PyMOL Molecular Graphics System was used to visualize the key interactions and point out differences in the WT and mutant structures (PyMOL Molecular Graphics System, version 2.0, Schrödinger, LLC).Interaction energies of the gating charges and the S4 helix to the residues in S1, S2 and S3 of VSD I were calculated using AmberTools23 (Case et al., 2023).

### Electrophysiology

Calcium currents were recorded with the patch-clamp technique with the cells being voltage-clamped in the whole cell mode. The patch pipettes (borosilicate glass, Harvard Apparatus, Holliston, MA) were prepared to yield a resistance of 2 - 4 MΩ when filled with (mM) 145 Cs-aspartate, 2 MgCl_2_, 10 HEPES, 0.1 Cs-EGTA, and 2 Mg-ATP (pH 7.4 with CsOH). The extracellular bath solution contained (mM) 10 CaCl_2_, 145 tetraethylammonium chloride, and 10 HEPES (pH 7.4 with CsOH).

The recordings were performed with HEKA EPC 10 USB amplifier (Harvard Bioscience Inc.). Data acquisition and command potentials were controlled by PATCHMASTER NEXT (version 1.2, Harvard Bioscience Inc.).

The cells were clamped at a holding potential (V_hold_) of −80 mV, followed by the command potential (V_cmd_) of 500ms, ranging from −60 mV to +80 mV with an increment of 10 mV. Before each sweep a leak subtraction pulse was applied with a duration of 10 ms at a potential of -120 mV. Between command potentials the cell was repolarized to the holding potential (-80 mV) for a duration of 250 ms. The current-voltage dependence was calculated according to

(1) $$I=G_{max}*(V-V_{rev\;})/(1+\exp(-\frac{V-V_{1/2}}{K})),$$

where G_max_ is the maximum conductance of the L-type calcium channels, V_rev_ is the extrapolated reversal potential of the current, V_1/2_ is the potential for half-maximal conductance, and K is the inverse of the slope.

The conductance voltage-dependence was calculated according to the Boltzmann distribution:

(2) $$G=G_{max}/(1+\exp(-\frac{V-V_{1/2}}{K})).$$

The mean ± SEM for the calcium currents (I_Ca_) sample traces was calculated by selecting the sweep at V_max_ for each recording constituting the dataset.

The time constant of activation (τ_Activation_) was evaluated be fitting the rising phase of the currents at V_max_ with a single exponential function.

### Statistics

Statistical analysis and curve fitting was performed using SigmaPlot (version 12.0.0.182, Grafiti LLC). Figures were prepared in GraphPad Prism (version 10.2.3, GraphPad Software LLC) and Affinity Designer and Photo (version 2.5.3, Serif (Europe) Ltd.). All data are represented as mean ± SEM or mean ± SD. Figures presenting current traces the point by point calculated mean ± SEM. When comparing two different data sets, the statistical fit parameters were obtained by using a Student’s t test. In case of non-Gaussian distribution of the data set, tested with a Shapiro-Wilk test, we performed a Mann-Whitney test. If the variances of the data set showed a significant difference, tested with a F-test, a Welch t-test was performed. To compare multiple data sets we performed one-way ANOVA combined with Dunnett’s multiple comparison post hoc test with significance criteria. In case of non-Gaussian distribution of the data set, tested with a Shapiro-Wilk test, we performed a Kurskal-Wallis test with Dunn’s multiple comparison post hoc test. If the variances of the data set showed a significant difference, tested with a Brown-Forsythe test, a Welch ANOVA test with Dunnett’s T3 multiple comparison post hoc test was performed. The significance criteria are as follows *p < 0.05, **p < 0.01, ***p < 0.001, and ****p < 0.0001. The exact p-values for all statistical tests are shown in Tables 1 and 2.

## Results

The transition pathway of the positively charged S4 helix through the VSD runs through an aqueous inner vestibule, containing the two conserved countercharges of the CTC in S2 and S3, across the HCS, consisting of the conserved phenylalanine in S2 plus additional hydrophobic residues in S1, S2 and S3, into the outer aqueous vestibule, containing various numbers of additional countercharges in different positions (Fig. 1F) (Catterall et al., 2017; Groome and Bayless-Edwards, 2020). The HCS is between 4 to 10 Å wide and encompasses the highly focused electric field across which the gating charges need to pass upon depolarization and repolarization (Ahern and Horn, 2005; Delemotte et al., 2011). Cryo-EM structures of voltage-gated calcium channels and X-ray structures of Na_V_Ab, the prokaryotic ancestor of Ca_V_ and Na_V_ channels, consistently show that in the up-state (at 0 mV) the inner gating charge (here designated R4) engages in the CTC just below the HCS (Wu et al., 2016; Payandeh et al., 2012; Gao et al., 2021, 2023; Zhao et al., 2019). In the resting state structure of the disulfide-linked Na_V_Ab the S4 helix has moved inward by two complete 3_10_ helical turns, with both R2 and R3 forming ion pairs with the countercharges of the CTC and only R1 remaining above the HCS. Resting state structures of Ca_V_ channels (or eukaryotic Na_V_ channels) are still elusive (but see (Gao et al., 2023, 2021; Yao et al., 2022)). Therefore, we performed MD simulations of Ca_V_1.1 in a membrane environment exposed to an electric field to describe the state transitions of its VSDs from the (in-)activated up-state to the resting state (Pelizzari et al., 2024).

Within approximately 3 µs simulation time all four VSDs of Ca_V_1.1 moved out of the known up-state into deeper intermediate or resting states, in a way consistent with the overall predictions of the sliding helix model (Fig. 1C, E). While the simulation time required for the downward movements and the exact positions of the final states achieved differed between the four VSDs, they all showed the sequential formation of ion pairs between the S4 gating charges and countercharges in S2 and S3. In the lowest down-state achieved within the >3 µs unconstrained MD simulations the S4 helices of VSDs I, II, III, and IVa all moved down by approximately two helical turns with the gating charges R3 and R2 consecutively crossing the HCS (Fig. 1C). In agreement with previous functional studies (Tanabe et al., 1991; Tuluc et al., 2016a; Fernández-Quintero et al., 2021), in the unconstrained MD simulation VSD I displayed the slowest movement of all four VSDs. Tracing the vertical displacement of the C_α_ atoms of the five IS4 gating charges relative to the position of F97 during the MD simulation shows two steps in the downward movement (Fig. 1E). Starting from the up-state (modeled from the cryo-EM structure of Ca_V_1.1 (Wu et al., 2016)), R3 rapidly moved from its position above the HCS down into the CTC just below the HCS, thus stretching the helix between R2 and R3 into a 3_10_ configuration. This intermediate state was maintained for >1000 ns simulation time, before R4 exited the CTC and R2 moved down across the HCS, establishing interactions with the CTC counter charges. During this second jump, the helix stretch moved up one notch so that during the entire simulation a segment of 3_10_ helix spanned the HCS with the gating charges tightly anchored in the ENC and CTC, above and below the HCS, respectively. The conformation assumed in this second step was stable for the remainder of the simulation time (up to 3 µs). Notably, in this state the structure of VSD I closely resembles the cryo-EM resting state structure of Na_V_Ab (PDB 6P6W)(Wisedchaisri et al., 2019) and of a Na_V_1.7/Na_V_Ab chimera trapped in a deactivated state (PDB: 6N4R)(Xu et al., 2019) (Fig. 1D). Fig. 1G shows representative structures of the VSD in the up-state, the intermediate state and the lowest down-state of VSD I, plus (below) simplified cylinder models depicting the gating charge – countercharge interactions in the three states. Together this evidence indicates that in our unconstrained MD simulation of Ca_V_1.1 VSD I has traveled in two steps from its experimentally determined up-state into a stable down-state.

The structures in Fig. 1G and the simplified cylinder models below highlight the ion-pair interactions formed by the gating charges in the consecutive states. While R2 and R3 cross the HCS and exchange ion-pair partners between the ENC and CTC, R1 and R4 remain in the extracellular and intracellular hydrophilic vestibules, respectively. Note that R4 engages in interactions with the CTC in the intermediate and the up-state. In the down-state R4 is below the gating pore and does not participate in the process. In contrast, R1 sequentially exchanges interactions with countercharges E90, E87 and E140 within the ECN. This dynamic pattern of consecutively formed interactions suggests that the involved ion-pair partners serve multiple distinct functions in the gating charge transfer across the membrane electric field (R2, R3) and in stabilizing the activated or resting states (all gating charges). Therefore, we hypothesize that the contributions of S4 gating charges to the regulation of gating properties depend on their capacity to cross the HCS or not, and on the states in which they interact with countercharges of the CTC and the ENC. Experimentally, the distinct roles of the gating charges and their countercharges in the voltage-sensing process shall be revealed by specific effects of individual charge-neutralizing mutations on the kinetics and voltage-dependence of current activation.

In a first set of mutagenesis experiments, we substituted each one of the five gating charges (K0, R1, R2, R3, R4) of Ca_V_1.1 VSD I with alanine and examined the effects on the gating properties of whole-cell calcium currents. All experiments were performed in the native expression system for Ca_V_1.1, myotubes differentiated from the GLT cell line of dysgenic mouse (Ca_V_1.1-null) skeletal muscle, transfected with GFP-coupled wildtype or mutant Ca_V_1.1e constructs. Double-immunostaining with antibodies against the type 1 ryanodine receptor (RyR1) and against GFP-Ca_V_1.1 demonstrated that the constructs were expressed at comparable levels and correctly targeted into plasma membrane-SR junctions (i.e. triads) (Suppl. Fig. 1). Throughout the study we used the embryonic splice variant Ca_V_1.1e, which activates at a half-maximal voltage between +5 and +10 mV and conducts several-fold larger calcium currents compared to the adult splice variant Ca_V_1.1a (Tuluc et al., 2009). Patch-clamp recordings were performed in multiple myotubes of at least three separate experiments and analyzed with matched controls of parallel transfections.

**Figure 2:**
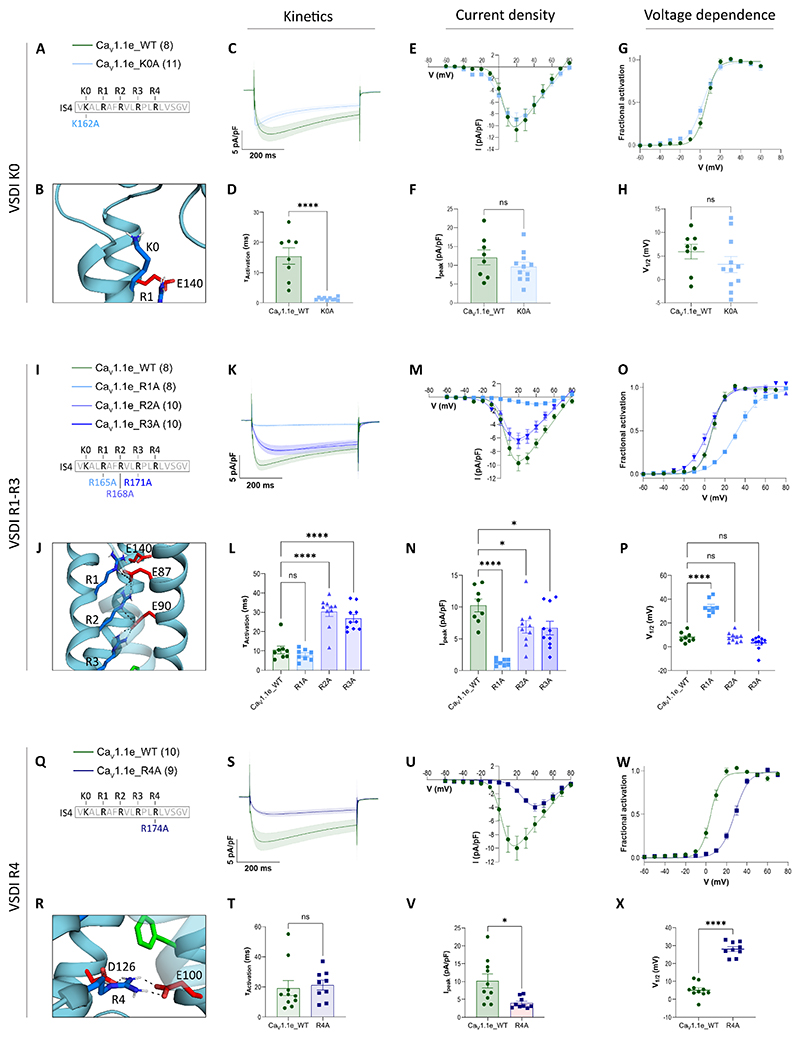
Gating properties of Ca_V_1.1e with individually mutated gating charges. Displayed are mean calcium currents, the I/V- and G/V-curves (±SEM) and scatter plots of the time constants of activation (τ_Activation_), maximal current density (I_peak_) and the voltage-dependence of activation (V_½_) of whole-cell patch-clamp recordings in dysgenic (Ca_V_1.1-null) myotubes reconstituted with wildtype (WT) and mutant Ca_V_1.1e. **A**: Legend and sequences indicating the positions of the mutated gating charges in the S4 helix of VSD I. **B**: Structure showing the position of K0 in the up-state. **C-H**: Alanine-substitution of K162 (K0A) accelerated activation kinetics. **I**: Legend and sequences indicating the positions of the mutated gating charges in S4. **J**: Structure showing the interactions of R1, R2 and R3 in the up-state. **K-P**: Individual alanine-substitutions of R165 (R1A), R168 (R2A) and R171 (R3A) right-shifted the voltage-dependence in R1A or decelerated the kinetics of activation in R2A and R3A. Current density was substantially reduced in R1A and slightly in R2A and R3A. Please note that in panels K and O the trace of R2A is almost hidden behind those of R3A and WT, respectively. **Q**: Legend and sequences indicating the position of the mutated S4 gating charge. **R**: Structure showing the interactions of R4 in the up-state. **S-X**: Alanine-substitution of R174 (R4A) right-shifted the voltage-dependence of activation and reduced peak current densities similar to R1A. For each mutation recordings from 8 – 11 (n) myotubes of at least three separate experiments (passages and transfections) were compared with parallel recorded controls. The data points are represented as mean ± SEM. Statistical tests: panels F, H and X, t-tests; panel V, Welch’s t-test; panels D and T, Mann-Whitney tests (K0, R4); panels L, N and P, one-way ANOVA with Dunnett’s multiple comparison (R1, R2, R3). ∗ ≜ *p <* 0.05, ∗∗ ≜ *p <* 0.01, ∗∗∗ ≜ *p <* 0.001, ∗∗∗∗ ≜ *p <* 0.0001. Exact p-values for all constructs are provided in Table 1.

In Ca_V_ channels, the S4 helices of all four VSDs contain the four gating charges R1, R2, R3 and R4. They correspond to the four gating charges found in the ancestral prokaryotic sodium channels (Fig. 1A) (Payandeh et al., 2011) and can be considered the prototypical set of gating charges. VSD I of the high-voltage activated Ca_V_1 and Ca_V_2 channels contains an additional gating charge at the extracellular end of the S4 helix (K162 or K0). Alanine-substitution of this residue (Ca_V_1.1e_K162A) had little to no effect on current density and voltage-dependence of activation. However, compared to wildtype controls, Ca_V_1.1e_K162A displayed a greater than ten-fold acceleration of activation kinetics measured as the time-to-peak (Fig. 2A-H, Table 1). In contrast, individual mutations of R1 (Ca_V_1.1e_R165A) and R4 (Ca_V_1.1e_R174A) both resulted in a striking reduction of the current density and in a >22 mV shift of the voltage-dependence of activation to more positive potentials (Fig. 2I-P, Q-X; Table 1). Activation kinetics were not significantly different from that of wildtype controls. Expression levels and co-clustering with RyR1 of the mutant constructs were comparable between myotubes transfected with the respective mutants and wildtype controls (Suppl. Fig. 2 and Table S1), indicating that the decreased current density resulted from altered voltage sensor function. Furthermore, individual mutations of R2 (Ca_V_1.1e_R168A) and R3 (Ca_V_1.1e_R171A) modestly reduced the current density to about 70% of control values. In variance to the mutations of K0, R1 and R4, the alanine substitutions of R2 and R3 slowed the activation kinetics about 2.5-fold and 3-fold, respectively, but did not shift the voltage-dependence of current activation (Fig. 2I-P; Table 1). Together, these experiments show that the functional effects of substituting the positively charged long-chain amino acids arginine or lysine with the small, uncharged alanine fall into two distinct groups: those affecting current density and the voltage-dependence of activation (R1, R4) and those specifically affecting activation kinetics (K0, R2, R3). The latter group further divides into the outermost gating charge K0, the presence of which slows activation, and the two middle gating charges, R2 and R3, which accelerate it. Thus, for the four prototypical gating charges (R1-R4), the decisive factor of the functionally distinct pairs appears to be, whether they cross the HCS (R2, R3) or not (R1, R4). The additional upper gating charge K0 appears to be critically involved in determining the characteristically slow activation kinetics of the skeletal muscle Ca_V_1.1 channel.

**Figure 3:**
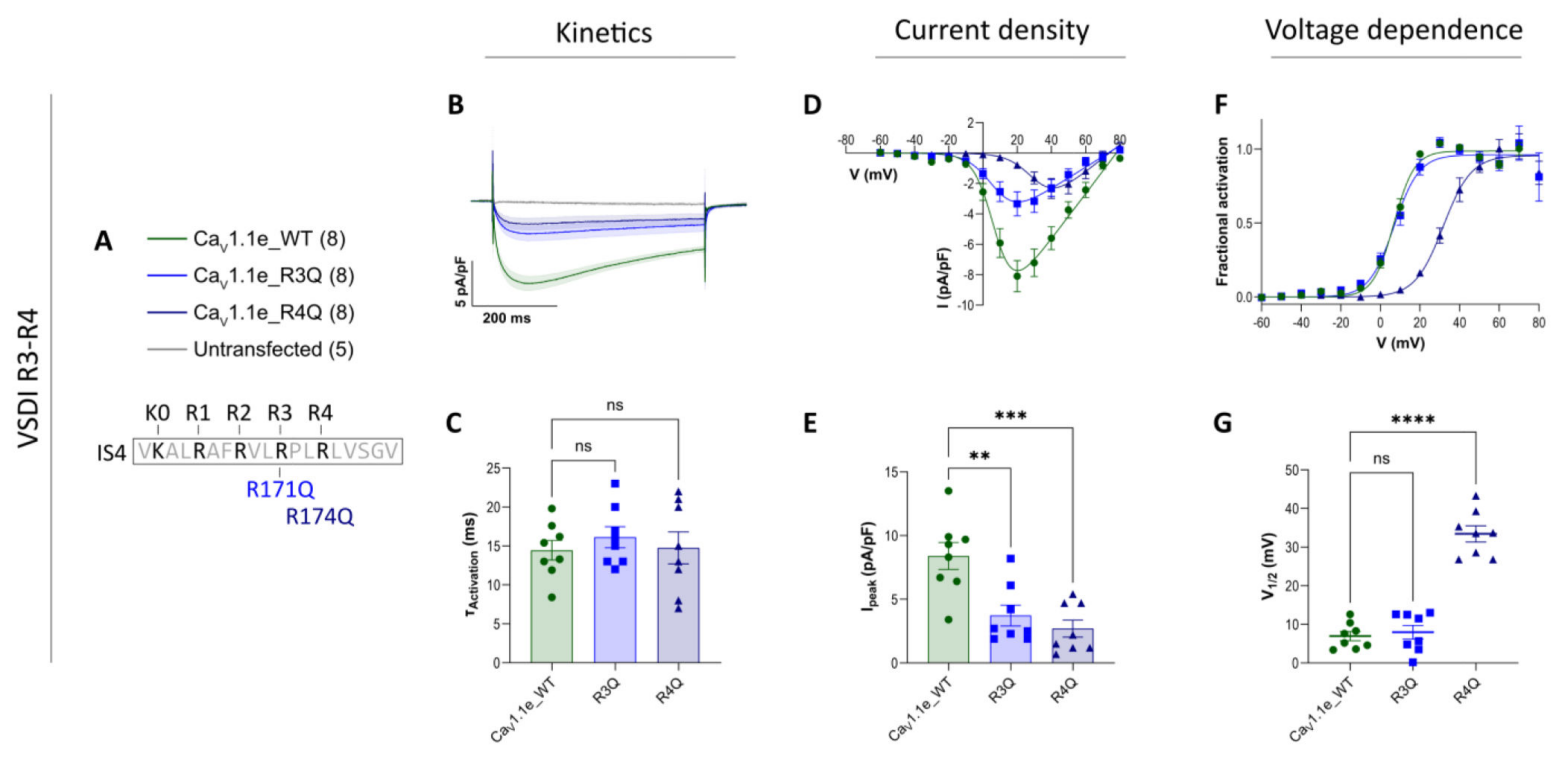
Gating properties of Ca_V_1.1e with glutamine substitutions of representative gating charges. Displayed are mean calcium currents, the I/V- and G/V-curves (± SEM) and scatter plots of time constants of activation (τ_Activation_), maximal current density (I_peak_) and the voltage-dependence of activation (V_½_) of whole-cell patch-clamp recordings in dysgenic (Ca_V_1.1-null) myotubes reconstituted with wildtype (WT) and mutant Ca_V_1.1e. **A**: Legend and sequence of IS4 indicating the positions of the mutated gating charges. **B-G** Glutamine-substitution of R174 (R4Q) right-shifted the voltage-dependence of activation and both individual glutamine-substitutions (R3Q and R4Q) reduced the peak current densities. Additionally, panel B shows the current trace of untransfected cells for comparison. For each mutation, recordings from 5-8 (n) myotubes of at least three separate experiments (passages and transfections) were compared with parallel-recorded controls. The data points are represented as mean ± SEM; mutants and wt control are compared by one-way ANOVA with Dunnett’s multiple comparison. ∗ ≜ *p <* 0.05, ∗∗ ≜ *p <* 0.01, ∗∗∗ ≜ *p <* 0.001, ∗∗∗∗ ≜ *p <* 0.0001. Exact p-values for all constructs are provided in Table 1.

Strictly speaking, the effects of alanine substitutions cannot be equated with neutralization of the gating charges. By replacing an arginine with an alanine also the size and hydrophobicity of the amino acid side chain are changed and these differences can similarly affect the transition from the resting to the activated state. To examine this possibility, we further analyzed the functional effects of substituting two of the gating charges with glutamine: Ca_V_1.1e_R171Q (R3Q) representing the HCS-passing gating charges, and Ca_V_1.1e_R174Q (R4Q) representing the non-passing gating charges. The length of the glutamine side chain lies in between those of alanine and arginine, and unlike arginine, glutamine is uncharged and polar, which excludes formation of ion bonds but still enables it to form hydrogen bonds with the countercharges. Interestingly, the current properties of Ca_V_1.1e_R171Q (R3Q) did not significantly differ from those of the wildtype control (Ca_V_1.1e) (Fig. 3B-C), indicating that positive charge and the ability to form ionic bonds with countercharges are not essential for the role of this gating charge in accelerating activation kinetics. In contrast, glutamine substitution of R4 (Ca_V_1.1e_R174Q) recapitulated the effects observed with R4A (Fig. 3F-G), indicating that its role in setting the voltage-dependence of activation indeed requires the formation of ionic bonds with countercharges.

**Figure 4:**
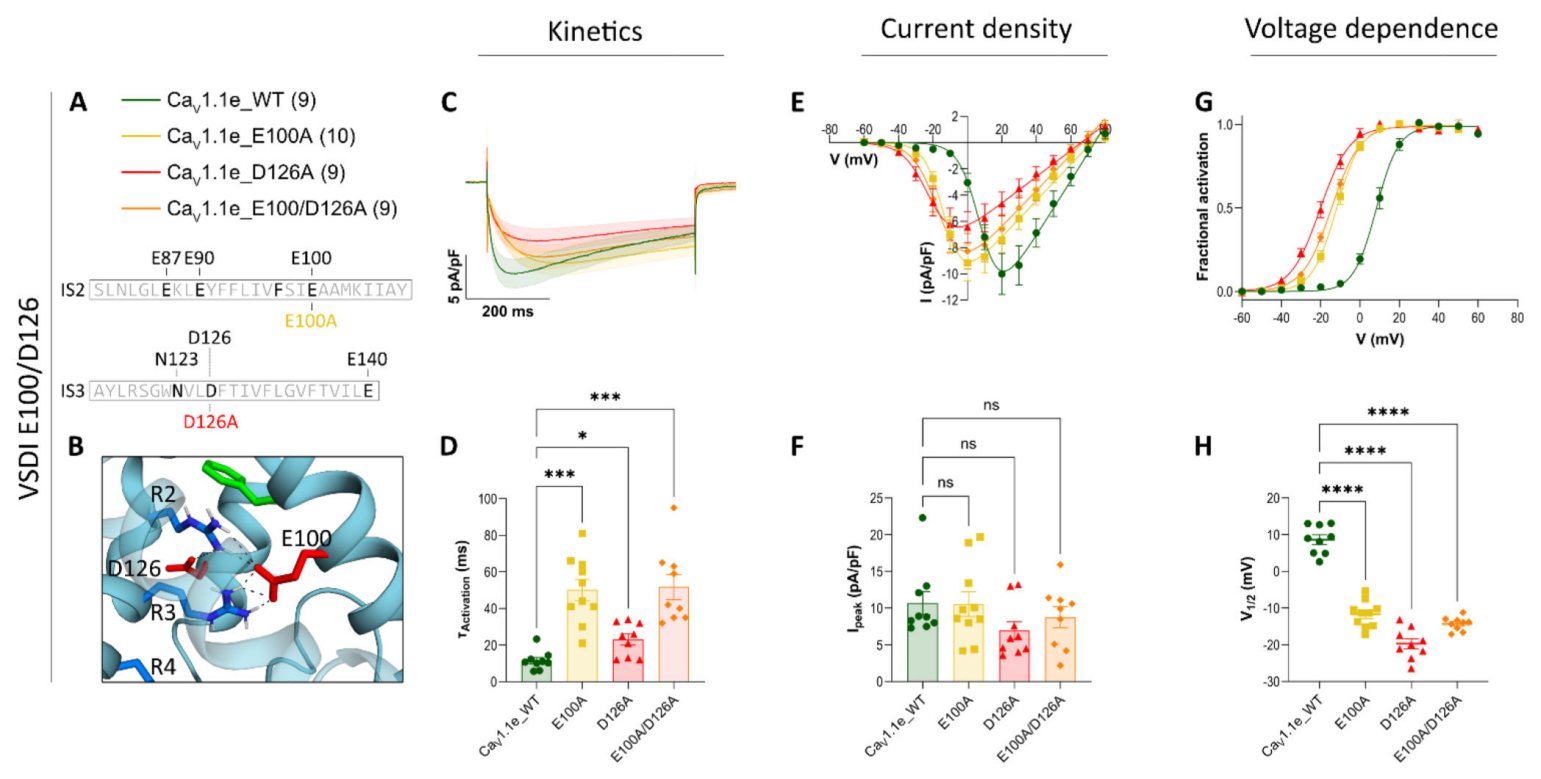
Gating properties of Ca_V_1.1e with mutated charge transfer center (CTC) countercharges. Displayed are mean calcium currents, the I/V- and G/V-curves (±SEM) and scatter plots of the time constants of activation (τ_Activation_), maximal current density (I_peak_) and the voltage-dependence of activation (V_½_) of whole-cell patch-clamp recordings in dysgenic (Ca_V_1.1-null) myotubes reconstituted with wildtype (WT) and mutant Ca_V_1.1e. **A**: Legend and sequence indicating the positions of the mutated countercharges in the S2 and S3 helices of VSD I. **B**: Structure showing the interactions of E100 and D126 in the down-state. **C-H**: Individual alanine-substitutions of E100 (E100A) and D126 (D126A) as well as the double mutation (E100A/D126A) left-shifted the voltage-dependence and decelerated the kinetics of activation to similar degrees. Current densities remained unaltered. For each mutation, recordings from 9 – 10 (n) myotubes of at least three separate experiments (passages and transfections) were compared with parallel-recorded controls. The data points are represented as mean ± SEM. Statistical analysis: panel D, one-way Welch-ANOVA with Dunnett’s T3 multiple comparison; panel F, one-way ANOVA with Dunnett’s multiple comparison; panel H, Kurskal-Wallis test with Dunn’s multiple comparison. ∗ ≜ *p <* 0.05, ∗∗ ≜ *p <* 0.01, ∗∗∗ ≜ *p <* 0.001, ∗∗∗∗ ≜ *p <* 0.0001. Exact p-values for all constructs are provided in Table 2.

A central proposition of the sliding helix model is that these effects of the gating charges on the channel´s gating properties depend on their interactions with negative countercharges in the surrounding helices. The two highly conserved countercharges comprising the CTC have been proposed to “catalyze” the energetically unfavorable transition of the gating charges across the HCS (Tao et al., 2010). If the observed slowing of current kinetics in Ca_V_1.1e_R168A (R2A) and Ca_V_1.1e_R171A (R3A) resulted from impairment or loss of this catalytic effect and if the formation of hydrogen bonds or ion pairs between the two middle gating charges (R2, R3) and the CTC is involved in this process, we expect that mutation of these countercharges results in similar effects on current kinetics as observed when mutating R2 or R3. To examine this hypothesis, we substituted the countercharges of the CTC (E100 and D126) individually with alanines. As predicted, both mutations, Ca_V_1.1e_E100A and Ca_V_1.1e_D126A, slowed the current kinetics by about 5- and 2.0-fold, respectively, compared to the wildtype control (Fig. 4C-D; Table 2). Their current density remained unaltered, but, surprisingly, their voltage-dependence of activation was significantly shifted to more negative potentials (Fig. 4E-H, Table 2). The slowing of the activation kinetics by the individual mutation of the CTC countercharges (E100 and D126), similar to that of the alanine substitution of the the middle gating charges (R2 and R3), supports a role of E100 and D126 in facilitating the gating charge transitions across the HCS. The observation that channel gating in Ca_V_1.1e_E100A and Ca_V_1.1e_D126A is not abolished, further demonstrates that a single countercharge in the CTC may be sufficient to exert its catalyzing effect on charge transfer, albeit at a reduced rate. To test this possibility, we examined whether the effects observed in the single mutations are additive, by analyzing the double mutant Ca_V_1.1e_E100A/D126A. However, the results indicate that this was not the case. The magnitude of the effects of the double mutation on the slowing of current kinetics and on left-shifting the voltage-dependence of activation was similar to that observed in the single Ca_V_1.1e_E100A mutation (Fig. 4C-H; Table 2). This indicates that the combination of both countercharges in the CTC is necessary to exert its full facilitating action on the transfer of R2 and R3 across the HCS. The fact that the CTC mutations did not abolish channel gating altogether, further suggests that the action of the CTC is merely facilitating, but not essential for the voltage-sensing action.

**Figure 5:**
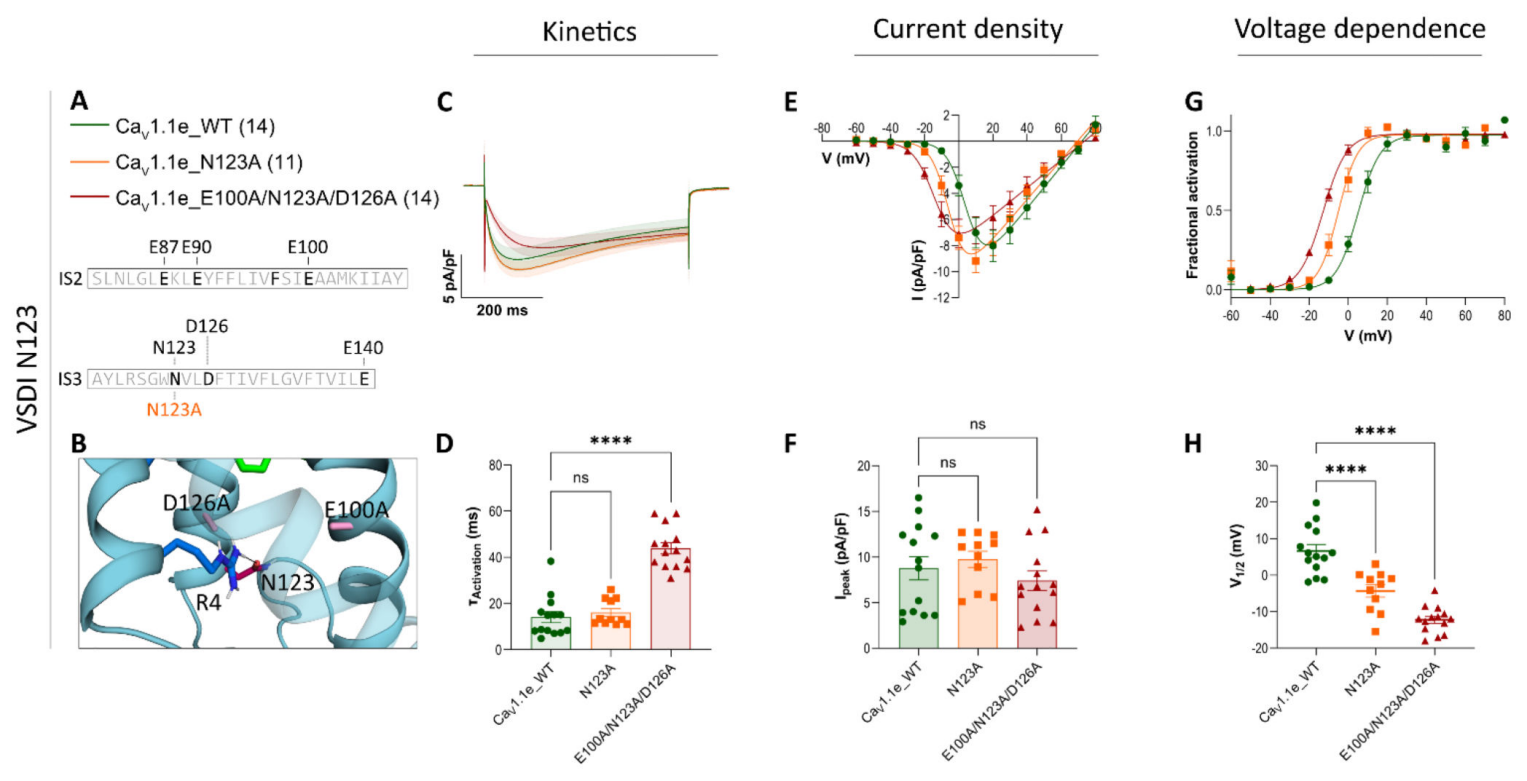
Gating properties of Ca_V_1.1e with mutated putative interaction partner N123 with and without mutations in the CTC. Displayed are mean calcium currents, the I/V- and G/V-curves (±SEM) and scatter plots of time constants of activation (τ_Activation_), maximal current density (I_peak_) and the voltage-dependence of activation (V_½_) of whole-cell patch-clamp recordings in dysgenic (Ca_V_1.1-null) myotubes reconstituted with wildtype (WT) and mutant Ca_V_1.1e. **A**: Legend and sequence indicating the positions of the mutated residues in the S2 and S3 helices of VSD I. **B**: Structure showing the interactions of N123 in the absence of E100 and D126 in the up-state. **C-H**: The individual alanine-substitution of N123 (N123A) left-shifted the voltage-dependence of activation without affecting current kinetics. The combined mutation E100A/N123A/D126A left-shifted the voltage-dependence and slowed the kinetics of current activation to a similar degree as mutations of the two CTC countercharges alone (cf. Fig. 3). Current densities remained unaltered. For each mutation, recordings from 11 – 14 (n) myotubes of at least three separate experiments (passages and transfections) were compared with parallel-recorded controls. The data points are represented as mean ± SEM. Statistical analysis: panel D, Kurskal-Wallis test with Dunn’s multiple comparison; panel F and H, one-way ANOVA with Dunnett’s multiple comparison. ∗ ≜ *p <* 0.05, ∗∗ ≜ *p <* 0.01, ∗∗∗ ≜ *p <* 0.001, ∗∗∗∗ ≜ *p <* 0.0001. Exact p-values for all constructs are provided in Table 2.

Nevertheless, it is still possible that in the absence of one or both E100 and D126, nearby residues might compensate for the loss of the primary actors in the CTC. In fact, our structure model indicated that the adjacent highly conserved asparagine (N123) also interacted with the gating charges, its amino group forming hydrogen bonds with R4 (Fig. 5B). Therefore, we examined whether N123 is an active component of the CTC and/or whether it might compensate for the loss of E100 and D126. Like the mutations of the classical CTC countercharges, Ca_V_1.1e_E100A and Ca_V_1.1e_D126A, the individual Ca_V_1.1e_N123A substitution shifted the voltage-dependence of activation to more negative potentials (Fig. 5C-H; Table 2). However, it did not show the effect of slowed activation kinetics. This differential effect of the mutation indicates that N123 is actively involved in the voltage-sensing process, but that, in the presence of intact CTC countercharges E100 and D126, it does not facilitate the gating charge transfer across the HCS. Also, in combination with substitutions of the two CTC countercharges (E100A/D126A) the substitution of N123A did not further increase their effects on Ca_V_1.1 gating properties. Ca_V_1.1e_E100A/N123A/D126A resulted in slowed activation kinetics and in a left-shifted voltage-dependence of activation with comparable magnitudes as Ca_V_1.1e_E100A/D126A or the two individual substitutions Ca_V_1.1e_E100A and Ca_V_1.1e_D126A (Fig. 5C-H; Table 2). Similarly, also the mutation of N123 in combination with each individual CTC countercharge (Ca_V_1.1e_E100A/N123A and Ca_V_1.1e_N123A/D126A) did not alter the current properties beyond the effects of each CTC mutation alone (Suppl. Fig. 3). Thus, the observed interactions between N123 and the gating charges neither contribute to the charge transfer facilitating action of the CTC, nor compensate for its loss in the E100A, D126A mutations.

**Figure 6:**
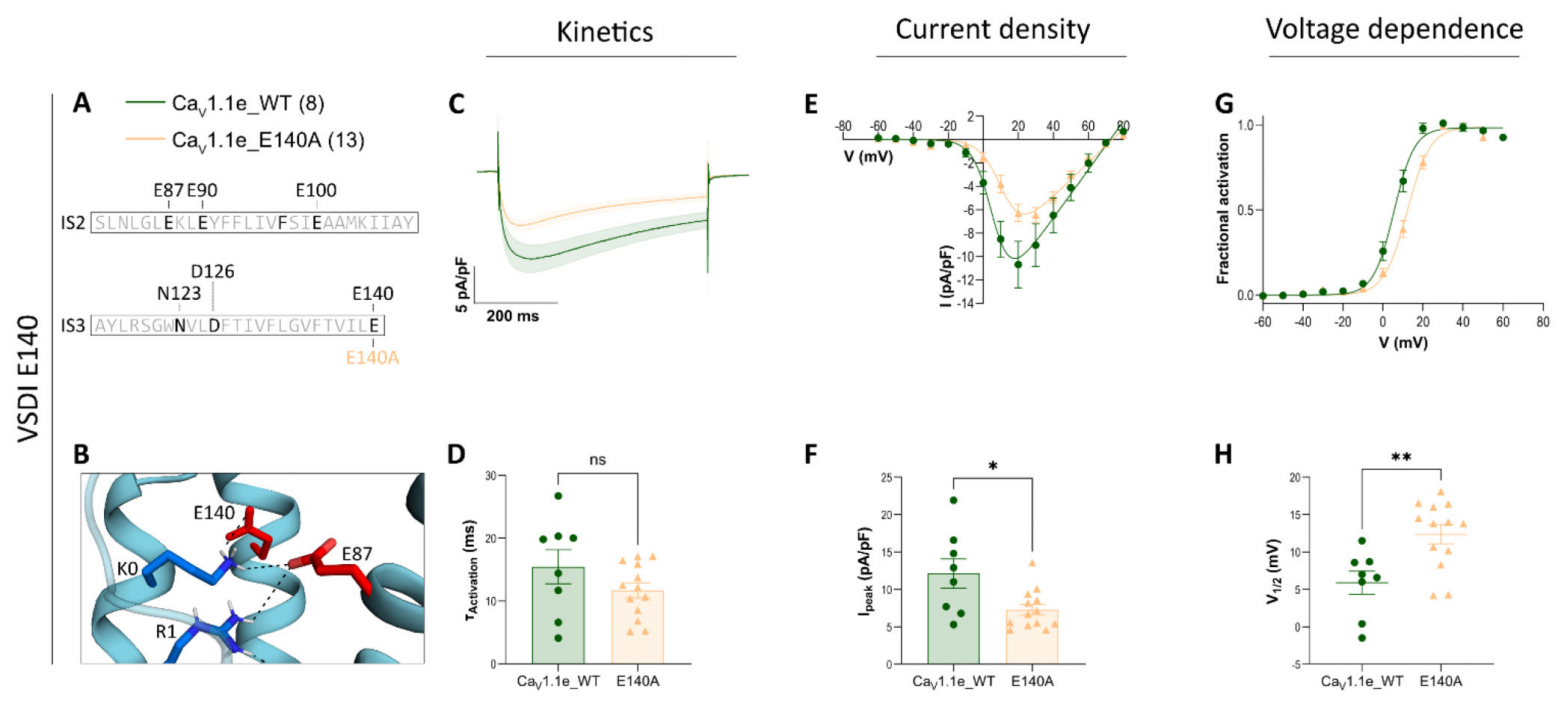
Gating properties of Ca_V_1.1e with the mutated putative countercharge E140 in the ENC. Displayed are mean calcium currents, the I/V- and G/V-curves (±SEM) and scatter plots of time constants of activation (τ_Activation_), maximal current density (I_peak_) and the voltage-dependence of activation (V_½_) of whole-cell patch-clamp recordings in dysgenic (Ca_V_1.1-null) myotubes reconstituted with wildtype (WT) and mutant Ca_V_1.1e. **A**: Legend and sequence indicating the position of the mutated countercharges at the extracellular end of the S3 helix of VSD I. **B**: Structure showing the interactions of E140 in the down-state. **C-H**: The individual alanine-substitution of E140 (E140A) slightly accelerated activation kinetics, reduced the current density and right-shifted the voltage-dependence of activation. For each mutation, recordings from 8 – 13 (n) myotubes of at least three separate experiments (passages and transfections) were compared with parallel-recorded controls. The data points are represented as mean ± SEM. Statistical analysis: panel D, t-test; panel F, Welch’s t-test; panel H, Mann-Whitney test. ∗ ≜ *p <* 0.05, ∗∗ ≜ *p <* 0.01, ∗∗∗ ≜ *p <* 0.001, ∗∗∗∗ ≜ *p <* 0.0001. Exact p-values for all constructs are provided in Table 2.

Previously we characterized the function of two countercharges of the ENC, E87 and E90, using site directed mutagenesis and found that they are differentially involved in regulating the voltage-dependence and kinetics of activation (Fernández-Quintero et al., 2021). Our current structure models predict additional transient interactions of the outermost gating charge K0 with another potential countercharge (E140) at the cytoplasmic end of the IS3 helix. Considering the observed acceleration of activation in K0A (Ca_V_1.1e_K162A, see above), formation and subsequent braking of the ionic interaction between K0 and E140 might contribute to this kinetic effect. To examine this possibility we substituted the putative countercharge with alanine (Ca_V_1.1e_E140A) and analyzed the gating properties of this channel mutant (Fig. 6). Ca_V_1.1e_E140A showed slightly accelerated activation kinetics, a modest, but significant reduction of current density and right shift of voltage-dependence of activation (Fig. 6C-H, Table 2). Thus, E140, together with the previously characterized E87 and E90, functions as countercharge for the outer gating charges and together they regulate the characteristic gating properties of Ca_V_1.1.

The observed differential effects on the voltage-dependence and kinetics of activation—right-shifted without slowing of kinetics in R1A and R4A, slowing or accelerating without a shift of V½ in R2A, R3A, and K0, respectively, left-shifted with concurrent kinetic slowing in E100A and D126A, left-shifted without effect on kinetics in N123A, right-shifted with concurrent acceleration in E140A—clearly indicate that regulation of voltage-dependence by the interactions between gating charges and countercharges is independent of their “catalytic” action on gating charge transfer. The right shift of the voltage-dependence of activation to more positive potentials when mutating R1 and R4 is consistent with a function of their interactions in stabilizing the activated state. As expected similar right shifts have been observed here with E140A and previously when mutating R1´s primary interaction partner E87 (Fernández-Quintero et al., 2021). However, the left shift observed here when mutating the interaction partners of R4 in the activated state, E100 and D126, does not follow the expected pattern. Instead, the effect on voltage-dependence when mutating these CTC countercharges indicates their involvement in stabilizing the resting states, which occurs when E100 and D126 sequentially interact with R2 and R3. If this interpretation is correct, the voltage-dependence of activation depends on the balance between interactions stabilizing the activated and interactions stabilizing the resting states.

**Figure 7:**
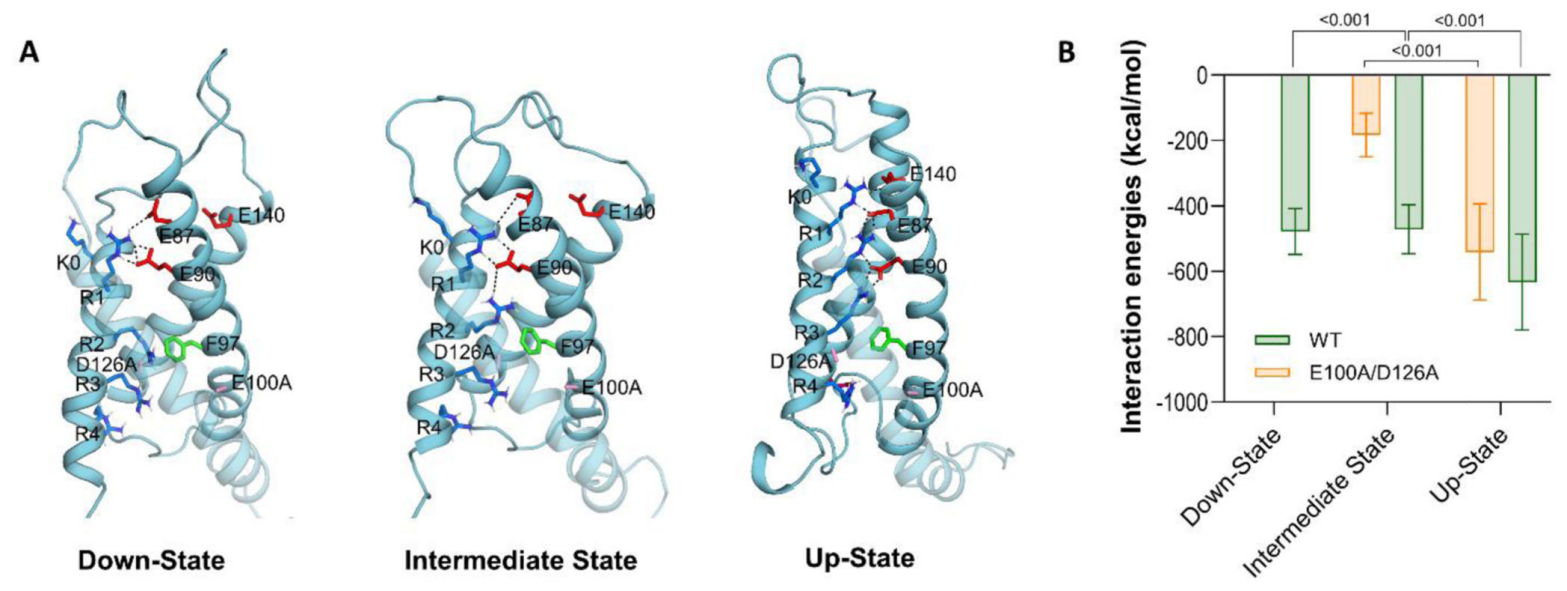
Interactions of S4 gating charges of wildtype and Ca_V_1.1e_E100A/D126A VSD I in all three states. **A**: Structures of Ca_V_1.1e_E100A/D126A VSD I in the down-, intermediate and up-states. Interactions between gating charges (blue) and countercharges (red) are formed outside but not inside the HCS indicated by F97 (green). **B**: Interaction energies of the VSD I S4 helix were calculated for the up-, intermediate and down-states of Ca_V_1.1e and Ca_V_1.1e_E100A/D126A. Abolished CTC interactions in Ca_V_1.1e_E100A/D126A increasingly destabilize the VSD as the S4 helix moves down. Mean ± SD; mutants and wt controls compared by t-tests.

To examine this concept, we interrogated the structures and calculated the interaction energies of the VSD I S4 helix in the consecutive states revealed in our MD simulation (Fig. 7). In wildtype Ca_V_1.1, the overall interaction energy of the S4 helix with its surroundings was higher in the up-state compared to the intermediate and down-states. This is expected, considering the propensity of the VSD to activate spontaneously in the absence of an electric potential pulling it into the energetically less favorable resting state. The structures in Figure 6A show how the double mutation of the two CTC countercharges (Ca_V_1.1e_E100A/D126A) changes the balance of gating charge interactions between the ENC and the CTC. In the up-state, only the interaction of R4 is lost in the E100A/D126A mutant, while R1, R2 and R3 still interact with the countercharges of the ECN. As S4 moves down, these interactions decline, but in the absence of the CTC no new interactions are established below the HCS. This is reflected by the drastic decline of IS4 interaction energies in the intermediate state and a complete destabilization of the down-state (Fig. 7B). Thus, removal of the CTC affects all states, but destabilization of the intermediate and down-states is much greater because of the greater importance of the CTC relative to the ENC in stabilizing the down-states of S4. Thus, neutralization of R4 specifically destabilizes the up-state, resulting in a right shift of V½, whereas neutralization of the CTC (E100A, D126A, N123A) predominantly destabilize the down-state, and thereby shift the balance of stabilizing interactions towards the activated/up-state, resulting in the observed left shift of V½ (cf. Figs. 3 and 4).

## Discussion

### The sliding helix model and its variations

The gating charges, structurally defined as the positively charged amino acids regularly spaced in the S4 helix, are the pivotal component of all VSDs. They are the points of action for the membrane electric field, pulling the S4 helix inward in the polarized resting state and outward when the membrane potential reverses during an action potential. They need to overcome the energy barrier comprised of the hydrophobic seal in the gating charge permeation pathway as they transition between the resting and activated states. And finally, the positive gating charges form multiple sequential ionic interactions with negatively charged countercharges on both sides of the HCS that are thought to facilitate the charge transfer across the hydrophobic seal and to stabilize the voltage sensor in the consecutive resting, intermediate and activated states. These elements embody the essence of the sliding helix model (Catterall et al., 2017). Beyond these universal features of VSDs, our current data suggest that the balance of these multiple activities of the gating charges determines the specific kinetics and voltage-dependence of voltage sensor actions and consequently shapes the distinct gating properties of voltage-dependent calcium channels.

### MD simulation reveals distinct movements of the four VSDs

Previously the voltage sensor movement of homotetrameric K_V_ channels have been studied using all-atom MD simulations of channels exposed to an electric field (Jensen et al., 2012; Delemotte et al., 2011). Here we utilized the first such MD simulation of a member of the Ca_V_/Na_V_ family of ion channels with a pseudo-heterotetrameric building plan to study the molecular mechanisms guiding the voltage sensor movement in a channel with four structurally similar but non-identical VSDs (Pelizzari et al., 2024). As expected, the movements of the four Ca_V_1.1 VSDs differed in kinetics, trajectories and molecular interactions established during the state transitions. Specifically, we focused on the VSD I, which is the chief determinant of Ca_V_1.1 gating properties (Savalli et al., 2021; Fernández-Quintero et al., 2021; Tanabe et al., 1991; Tuluc et al., 2016a), and identified the interactions between the S4 gating charges and countercharges in the S2 and S3 helices formed in consecutive states. Whereas the sequential exchange of interaction partners, as S4 slides down from the up- to the down-state, resembles the predictions of the sliding helix model, the gating charge interactions display important discriminations. Consistent with earlier findings in sodium and potassium channels (Yang et al., 1996; Ahern and Horn, 2004; Starace and Bezanilla, 2001), in Ca_V_1.1 VSD I only two of the gating charges (R2, R3) cross the HCS, whereby they exchange ion pair partners between the ENC (E87, E90, E140) and the CTC (E100, D126). Interactions with the highly conserved countercharges of the CTC (E100, D126) are uniform among the consecutive gating charges (R3, R2) and between different VSDs. In contrast, gating charge interactions with the variable countercharges of the ENC (in VSD I E87, E90 and E140) are divergent between the four VSDs. During the outward movement of S4, the weight of interactions with the ENC increases. In addition, in the up-state, R4 becomes locked in the CTC and thus further contributes to the stabilization of this state. Conversely, the pivotal roles of R1 and R4 in stabilizing the activated state is reflected in strongly reduced interaction energies between S4 and the other helices in the up-state of the R1A and R4A mutants. Such a strong stabilization of the up-state by the concomitant formation of these two ion pairs is consistent with the notion that in the absence of the inward pull of the negative membrane potential at rest, the energetically favorable state is in the up-position, causing the passive outward movement of S4 at depolarized membrane potentials. Together the structural information gained from our unbiased MD simulation reveals molecular details underlying sliding helix mechanism and suggests differential roles of individual gating charges and countercharges in this process.

### The uncertain nature of the up-state

An ambiguity related to the MD simulation is that the functional state of its starting structure is uncertain. Because the biochemical preparation of channels for cryo-EM structure analysis collapses the membrane potential to zero, the Ca_V_1.1 structure likely represents an inactivated state of the channel with the VSDs in the up-position and the pore closed (Wu et al., 2016). Typically, the up-state is equated to the activated state of the VSD, even if the channel is inactivated by as of yet unknown mechanisms independent of further charge movement across the membrane. However, in skeletal muscle fibers persistent depolarization has been demonstrated to interconvert the Ca_V_1.1 voltage sensor into a second mode, called charge 2 (Q2). This mode is characterized by the uncoupling of voltage sensor movement from channel gating resulting in slow inactivation and by a substantially left-shifted voltage-dependence of gating currents (Gregorio et al., 2017; Brum and Rios, 1987). If in our MD simulation VSD I S4 moved along this path (mode 2), its trajectory and molecular interactions might differ from the state transitions taken on activation (mode 1), thereby creating a possible mismatch between our structures and electrophysiological analyses. However, to date the structural basis of the charge interconversion is unknown and there is no experimental evidence that it involved structural differences within the VSDs. Nor do we know whether and to what extent the four structurally and functionally distinct VSDs of Ca_V_1.1 participate in charge interconversion. In fact, given its inherently slow activation kinetics (100’s of ms), the charge movement of VSD I probably has not even been captured in the brief gating currents. Eventually, new experimental structures and extended MD simulations in the activating direction may shed light on this issue. In the absence of such evidence, we have to work on the simplest assumption that the structures of the MD simulation in the deactivating direction adequately represent the state transitions in the activating direction. Unfortunately, the analysis of current deactivation properties does not solve this problem either, because channel closure is controlled by the VSD with the fastest kinetics and most right-shifted voltage-dependence, which in Ca_V_1.1 is not VSD I.

### HCS-passing and non-passing gating charges:

**Figure 8:**
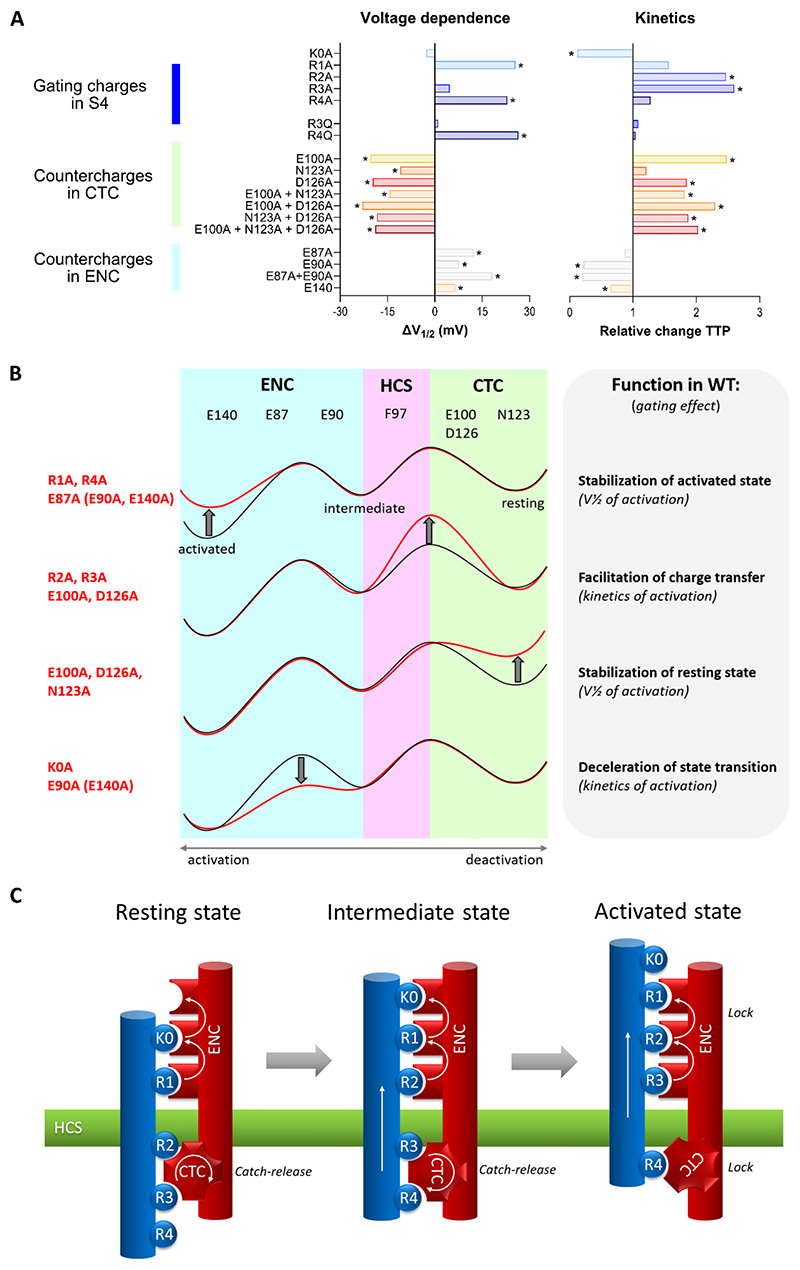
Energetic and mechanistic models illustrating the differential functions of gating charge – countercharge interactions in VSD state transitions. **A**: Summary of the effects on voltage-dependence and kinetics of activation of alanine-substitutions of gating charges and countercharges in Ca_V_1.1 VSD I. E87A and E90A data are from (Fernández-Quintero et al., 2021). **B**: Reaction energy profiles of the VSD charge transition pathway lined by basic counter charges in the extracellular cluster (ENC), and the charge transfer center (CTC) on both sides of the hydrophobic constriction site (HCS). Red traces demonstrate how charge-neutralizing mutations in the different domains alter energy barriers and energy wells to change the kinetics and voltage-dependence of activation, respectively. The three states and the energy barriers in between have been derived from our MD simulation (Fig. 1E, G). **C**: Mechanistic model of sequentially changing gating charge – countercharge interactions upon VSD state transitions. As S4 moves from the resting (down-) state through the intermediate state into the activated (up-) state, R2, R3 and R4 sequentially engage in the CTC and K0 to R3 in the ENC. Only R2 and R3 fully move across the HCS. The propensity of R4 to establish strong interactions with the CTC in the activated state may push R2 and R3 out of the CTC and across the energy barrier of the HCS, thus accelerating activation. On the other hand, the sequential occupancy of the ENC countercharges by the outer gating charges increases stabilization of the activated state, while decreasing the speed of activation. As the number and nature of counter charges in the ENC differs considerably between VSDs, this difference will confer distinct gating properties upon different channels.

Fig. 8A summarizes the bimodal effects on kinetics and voltage-dependence of activation of the charge-neutralizing mutations analyzed here and in a previous study (Fernández-Quintero et al., 2021). Studying the characteristically slow VSD I of Ca_V_1.1, we found that substituting the two gating charges that pass the HCS (R2A, R3A), or substituting the two countercharges of the CTC that interact with them as they pass the HCS (E100A, D126A), all result in a similar slowing of activation kinetics. This supports the notion that formation of ion-pairs between gating charges and the CTC “catalyzes” the charge transfer across the HCS (Tao et al., 2010). Our additional observation that substitution of R3 with glutamine (R3Q) shows no kinetic effect indicates that, at least for this gating charge transition, an ionic bond is not essential. Instead, a weaker hydrogen bond interaction formed between (R3Q) acting as hydrogen bond donor and the acceptors of the CTC appears to be sufficient for catalyzing this step in the state transition.

Of course, this catalyzing effect of the CTC pertains only to the two gating charges that actually pass the hydrophobic seal, R2 and R3. On the other hand, our data indicate that interactions of all gating charges and countercharges sequentially stabilize the consecutive states as the VSD moves between the resting and activated state. Depending on which states are stabilized by the formation of a specific ion-pair, severing the interaction by substituting one or the other interaction partner with alanine, leads to a right- or left shift of the voltage-dependence of activation. Right shifts, as in R1A and R4A as well as in E140A and E87A and E90A (Fernández-Quintero et al., 2021), indicate that the dominating role of the ion pairs formed between these gating- and countercharges is in stabilizing the activated state. Substitution of R4 with glutamine (R4Q) caused an equal right-shift, indicating that indeed an ionic bond is required for this activated state-stabilizing action. Left shifts, as in E100A, N123A and D126A, indicate a dominating role of the ion pairs formed by these CTC countercharges in stabilizing the resting states, i.e. making it harder for the S4 helix to enter the activated state. This model is in line with the gating charge – countercharge interactions observed in the structures derived from our unbiased MD simulation (Fig. 1G) as well as with the calculated interaction energies (Fig. 6). Differential effects of gating charges stabilizing the activated and resting states have been observed previously in another L-type calcium channel. In VSD I of Ca_V_1.2 the presence of only the innermost gating charge (R4) is sufficient to activate the channel, with a left-shifted V½ compared to wildtype (Beyl et al., 2016). This is consistent with the important role of R4 in stabilizing the activated state when it engages in the CTC. Adding the next gating charge, R3, right-shifted the voltage-dependence of activation to wildtype values, indicative of its balancing role in the stabilization of resting and activated states. Together, our findings demonstrate that the sequentially formed interactions between gating charges and countercharges serve two independent functions—facilitation of charge-transfer and stabilization of states—which translate into regulation of activation kinetics and voltage-dependence, respectively. Moreover, voltage-dependence of activation is regulated by the balance of interactions stabilizing the resting and the activated states.

### Charge transfer center

The findings presented here also extend the CTC concept as originally formulated for VSDs of potassium channels (Tao et al., 2010). We demonstrate that the ionic interactions of the CTC not only facilitate the transition of the gating charges across the HCS, but at the same time participate in the stabilization of the activated and resting states. While the former function increases the speed of activation, the latter affects the voltage-dependence of activation in a state-dependent manner. In the activated state, R4 reaches the CTC and becomes locked in it without passing the HCS. This strongly contributes to the stabilization of the activated state, i.e. left-shifting the voltage-dependence without affecting activation kinetics. In contrast, transient interactions of the CTC countercharges with gating charges R2 and R3 not only facilitate their transition across the HCS (increasing kinetics) but at the same time stabilize resting states, thus right-shifting the voltage-dependence of activation. Intriguingly, our structure models indicated the participation of hydrogen bonds between gating charges and N123 in the action of the CTC. The observed left shift of the voltage-dependence of activation in Ca_V_1.1e_N123A suggests that this interaction does contribute to the stabilization of the resting states, in the same way as the canonical CTC countercharges E100 and D126. However, the lack of a kinetic effect in the N123A mutant demonstrates that N123 does not contribute to the facilitation of the gating charge transition across the HCS and thus is not part of the operative CTC. Interestingly, the kinetic effects of mutating E100 and D126 are neither comprehensive nor additive. Neutralizing E100 or D126 slows activation kinetics more than two-fold, which is consistent with its “catalyzing” action in charge transfer. The observation that simultaneously neutralizing both countercharges did not further slow down activation kinetics, indicates that the simultaneous actions of both countercharges are essential for achieving the full catalytic activity of the CTC. Moreover, the fact that charge transfer still works without the help of this highly conserved structure of VSDs demonstrates that the energy barrier imposed by the HCS on the movement of R2 and R3 across the electric field is not preventively high.

### Countercharges of the extracellular negatively charged cluster (ENC)

As opposed to the highly conserved countercharges constituting the CTC, the functions of the variable countercharges of the ENC are poorly defined (Groome and Bayless-Edwards, 2020). For VSD I the critical role of E87 and E90 in determining the specific gating properties of Ca_V_1.1 has been addressed in a previous study (Fernández-Quintero et al., 2021). Here we discovered the functional relevance of another negatively charged amino acid (E140) at the cytoplasmic end of IS3. The previously characterized E87 and E90, located at the extracellular end of the S2 helix, stabilize the activated state by interacting with R1 and R2. Their mutation (E87A/E90A) right-shifted the voltage-dependence of activation and thus mirrors the effect of mutating R1 shown in the present study. E140 may further contribute to this action, as its mutation (E140A) also caused a modest right shift of the voltage-dependence of activation. This, however, did not involve the interaction with K0, because in the up-state this gating charge has moved beyond the ENC and its mutation (K0A) did not alter V½.

On the other hand, E90, located one helical turn below E87, was identified as a key determinant of the slow activation kinetics of Ca_V_1.1 (Fernández-Quintero et al., 2021). When mutated (E90A), the current activation accelerated by more than five-fold compared to the wildtype control. Here we show that mutation of K0 similarly accelerated the activation kinetics. Thus, E90 and K0 are the chief determinants of the characteristically slow activation kinetics of the skeletal muscle calcium channel. However, our MD simulation does not indicate a direct interaction of these two residues. Instead, during the transition from the down- into the up-state both residues form several sequential interactions with multiple ion-pair partners, which is likely to slow down the transition process. Together, the manifold functionally relevant interactions between the outer gating charges and countercharges are consistent with the notion that the ENC represents the major regulatory means determining the specific gating properties of Ca_V_ channels.

The kinetic effects of E90 and to a smaller degree E140 are in the opposite direction than the kinetic effect observed by mutating the countercharges of the CTC. While the interactions within the CTC, between R2, R3 and E100, D126, accelerate activation by facilitating the transitions of the gating charges across the HCS, E90 and E140 form transient interactions in the aqueous part of the gating pore, which apparently slow down the final transition into the activated up-state. This suggests another distinguishing principle of the action of gating charge interactions. The effects on activation kinetics of the sequential formation and breaking of ionic interactions depend on the environment in which they occur. In the hydrophobic environment of the HCS, formation of such ion pairs or hydrogen bonds speeds up the movement of S4, probably by decreasing the energy barrier imposed by the hydrophobic seal. In the hydrophilic environment of the outer vestibule, such interactions slow down the movement of the S4 helix, probably by creating additional energy barriers, as ionic interactions are repeatedly formed and broken. Since the countercharges of the ENC vary considerably among the four VSDs of Ca_V_ channels the degree to which such transient ion-pair formation is realized, might regulate the kinetics of VSD state transitions and consequently the channel gating properties. In this context it is important to note that the countercharges studied here are fully conserved across the members of the Ca_V_ channel family. Therefore, the striking differences in activation properties observed among Ca_V_ channels need to be encoded in the non-conserved sequences of the VSDs. Nevertheless, these differences likely translate into distinct interactions between gating charges and the conserved countercharges. Our alanine substitution experiments demonstrate, which of these interactions function in regulating kinetics and voltage-dependence of activation in one or the other direction, and they suggest testable hypotheses for the underlying molecular mechanisms.

### Analysis of current properties vs. gating currents

The direct electrophysiological readout of VSD action is the analysis of gating currents, i.e. the capacitive currents associated with the movement of the positively charged S4 residues across the membrane electric field (Bezanilla, 2018). In homoterameric K_V_ and prokaryotic Na_V_ channels the four VSDs move in unison and charge-neutralizing mutations affect all four VSDs, resulting in fairly robust signals and relatively clear interpretation. In pseudo-heterotetrameric eukaryotic Ca_V_ and Na_V_ channels, however, the four VSDs can move with distinct kinetics and voltage-dependencies, and most point mutations within a specific VSD seem to affect specifically the activation properties of that particular VSDs. Four VSDs responding to different voltages and with different kinetics results in complex gating currents and inevitably makes the analysis of charge-neutralizing mutations more difficult. Specifically, VSD I is responsible for the extremely slow activation of Ca_V_1.1 (over hundreds of milliseconds) (Fernández-Quintero et al., 2021; Savalli et al., 2021). Therefore, its contribution to gating charge probably is not even evident in the typical recordings of the ON or OFF gating currents.

Instead, we utilized the analysis of current properties as a proxy of VSD I action. The observed bimodal modulation of activation kinetics (slowing with R2A, R3A, E100A, D126A vs. acceleration with K0, E90A and E140) as well as the bimodal modulation of the voltage-dependence (right-shifted in R1A, R4A, E87A, E90A, E140A vs. left-shifted in E100A, N123A, D126A) demonstrate that changes in VSD I function directly translate into corresponding changes of channel gating properties. For example, if we accept that the slowest of the four VSDs will limit the rate of activation, mutations in VSD 1 slowing activation kinetics (R2A, R3A, E100A, D126A) indicate that the mutated VSD is the slowest of the four CaV1.1 VSDs and the kinetics of current activation reflect the speed of VSD I activation. Conversely, finding mutations in VSD I which accelerate activation (K0A, E100A, E140A) indicates that for the kinetic range observed, this VSD has been the slowest. What we cannot determine unambiguously is that the fastest activation kinetics observed in these mutations resembles the actual speed of VSD I activation, which, theoretically, could be even faster if now another VSD had become rate limiting. Moreover, observing this bimodal regulation of current properties by mutations of gating- and countercharges in VSD I, is an impressive demonstration of the dominating role of VSD I in gating the Ca_V_1.1 channel, in line with results from voltage-clamp fluorometry experiments (Savalli et al., 2021). Thus, the concurrent action of other VSDs may modify the control of VSD I on the gating properties, however, evidently they do not mask the effects of mutations in Ca_V_1.1 VSD I in either direction.

## Conclusion

Both our MD simulations of Ca_V_1.1 VSD I transitioning from the up- to the down-states as well as the results of the mutagenesis experiments are consistent with the sliding helix model of VSD action. We see the S4 helix moving downward relative to the other three helices, with two of its gating charges (R2 and R3) crossing the HCS and all gating charges sequentially forming and braking ionic interactions with multiple countercharges in the S2 and S3 helices. The effects on gating properties of charge-neutralizing mutations of the gating charges and countercharges vis-à-vis the structure information allow us to deduce mechanistic principles guiding VSD function (Fig. 7B). The transient ion-pair formation has two separable effects on the kinetics and voltage-dependence of activation. The former reflects the sequential formation and braking of the ionic bonds that affects state transitions (i.e. the height of the energy barriers) and manifests in activation kinetics. The latter reflects the stabilization of states (i.e. the depth of the energy wells) by varying numbers of ionic bonds manifesting in the voltage-dependence of activation. Importantly, the direction of the effects of both these functions depends on the condition in which the ion-pair interactions occur. Exchange of ion pairs in the hydrophobic environment of the CTC reduce the energy barriers and accelerate activation, whereas in the hydrophilic environment of the aqueous vestibule they form energy barriers slowing activation. Ion pairs stabilizing the activated/up-state form energy wells causing a left shift of the voltage-dependence of activation, whereas when stabilizing the resting states (again by creating energy wells) they right shift the voltage-dependence of activation. Together, these functions of the gating charge – countercharge interactions readily explain the functional properties of VSD I in regulating Ca_V_1.1 channel gating. Considering the highly conserved building plan of VSDs, these mechanisms may well represent general principles for tuning the activation properties of VSDs to the specific requirements of their respective channels.

## Supplementary Material

Supplementary Materials

## Figures and Tables

**Table 1:** Biophysical parameters of whole-cell calcium currents of wildtype and gating charge mutants of Ca_V_1.1e

Constructs	N^1^	n^1^	I_Ca_ (pA/pF)	P-value	Δ	V_1/2_ (mV)	P-value	Δ	TTP (ms)	P-value	Δ	τ_Activation_ (ms)	P-value	Δ
Ca_V_1.1e	3	8	-12.1 ± 2.0	0.277	2.5	5.9 ± 1.5	0.273	2.7	86.5 ± 11.7	<0.0001	75.9	15.4 ± 2.7	<0.0001	15.9
Ca_V_1.1e_K162A	3	11	-9.7 ± 1.2			3.2 ± 1.7			10.5 ± 0.9			1.4 ± 0.1		

Ca_V_1.1e	4	8	-10.2 ± 1.0	<0.0001	9.0	8.0 ± 1.5	<0.0001	25.5	54.7 ± 10.1	0.247	31.0	10.5 ± 2.0	0.732	2.6
Ca_V_1.1e_R165A	6	8	-1.3 ± 0.2			33.6 ± 2.2			85.6 ± 18.0			7.9 ± 1.0		
Ca_V_1.1e_R168A	5	10	-6.9 ± 1.0	0.045	3.4	8.0 ± 1.2	>0.9999	0.0	135 ± 8.3	0.0002	80.4	30.4 ± 2.5	<0.0001	19.9
Ca_V_1.1e_R171A	6	10	-6.7 ± 1.1	0.032	3.6	3.3 ± 1.8	0.138	4.7	142.2 ± 13.0	<0.0001	87.6	26.9 ± 2.0	<0.0001	16.4

Ca_V_1.1e	6	10	-10.1 ± 1.9	0.010	6.1	5.2 ± 1.3	<0.0001	22.9	90.6 ± 17.7	0.339	25.2	19.2 ± 5.0	0.305	2.0
Ca_V_1.1e_R174A	4	9	-4 ± 0.5			28.1 ± 1.3			115.7 ± 18.5			21.1 ± 3.2		

Ca_V_1.1e	3	8	-8.4 ± 1.1	0.0019	4.7	7.0 ± 1.2	0.8834	1.0	84.8 ± 5.4	0.6944	7.3	14.5 ± 1.3	0.6811	1.7
Ca_V_1.1e_R171Q	3	8	-3.7 ± 0.8			8.0 ± 1.8			92.0 ± 7.7			16.1 ± 1.3		
Ca_V_1.1e_R174Q	3	8	-2.7 ± 0.7	0.0003	5.7	33.4 ± 2.1	<0.0001	26.4	88.2 ± 13.5	0.9614	3.6	14.9 ± 2.0	0.987	0.3

1 N, number of experimental repeats (cell passages and transfections); n, number of recordings

**Table 2:** Biophysical parameters of whole-cell calcium currents of wildtype and countercharge mutants of Ca_V_1.1e

Constructs	N^1^	n^1^	I_Ca_ (pA/pF)	P-value	Δ	V_1/2_ (mV)	P-value	Δ	TTP (ms)	P-value	Δ	τ_Activation_ (ms)	P-value	Δ
Ca_V_1.1e	5	9	-10.7 ± 1.6	1.000	0.1	8.7 ± 1.3	<0.0001	20.3	71.2 ± 11.8	0.0001	105.6	11.6 ± 1.7	0.0001	38.9
Ca_V_1.1e_E100A	7	10	-10.5 ± 1.7			-11.7± 1.1			176.8 ± 20.7			50.3 ± 5.8		
Ca_V_1.1e_D126A	4	9	-7 ± 1.2	0.218	3.7	-19.7 ± 1.4	<0.0001	28.3	131.6 ± 12.7	0.031	60.5	23.0 ± 3.0	0.016	11.6
Ca_V_1.1e_E100A/ D126A	6	9	-8.8 ± 1.4	0.703	8.8	-14.3 ± 0.6	<0.0001	22.9	163.4 ± 15.1	0.001	92.3	51.8 ± 6.9	0.001	40.2

Ca_V_1.1e	7	14	-8.8 ± 1.3	0.777	1.0	6.6 ± 1.8	<0.0001	11.0	73.5 ± 8.6	0.315	15.7	14.0 ± 2.4	>0.9999	2.0
Ca_V_1.1e_N123A	3	11	-9.8 ± 0.9			-4.3 ± 1.7			89 ± 4.5			16.0 ± 1.7		
Ca_V_1.1e_E100A/ N123A/D126A	5	14	-7.4 ± 1.1	0.589	1.4	-12.2 ± 1	<0.0001	18.9	148.9 ± 9	<0.0001	75.5	43.8 ± 2.4	<0.0001	29.8

Ca_V_1.1e	3	9	-10.8 ± 1.4	0.965	0.4	6.7 ± 1.3	<0.0001	14.3	82.5 ± 11.4	0.0009	67.2	15.4 ± 3.0	<0.0001	43.4
Ca_V_1.1e_E100A/ N123A	4	9	-10.4 ± 1.8			-14.3 ± 1.8			149.7 ± 9.5			43.4 ± 2.3		
Ca_V_1.1e_N123A /D126A	4	10	-6.4 ± 0.7	0.047	4.4	-18.3 ± 1.3	<0.0001	18.3	153.4 ± 13	0.0004	71.0	31.2 ± 5.4	0.0149	31.3

Ca_V_1.1e	3	8	-12.1 ± 2.0	0.014	4.8	5.9 ± 1.5	0.005	6.5	86.5 ± 11.7	0.012	30.4	15.4 ± 2.7	0.164	3.8
Ca_V_1.1e_E140A	4	13	-7.3 ± 0.7			12.4 ± 1.3			56.0 ± 4.8			11.7 ± 1.2		

1 N, number of experimental repeats (cell passages and transfections); n, number of recording

## Data Availability

The datasets generated and analyzed during the current study are available from the corresponding author upon reasonable request.

## Summary

The movement of gating charges across the membrane electric field in response to depolarization is the essence of the voltage-sensing mechanism gating voltage-dependent ion channels. Here the authors unravel molecular mechanisms in a voltage-sensing domain, which govern the kinetics and voltage-dependence of calcium channel activation.

## Abbreviations

Ca_V_: voltage-gated calcium channel
CTC: charge transfer center
ENC: extracellular negatively charged cluster
HCS: hydrophobic constriction site
Na_V_: voltage-gated sodium channel
RyR1: type 1 ryanodine receptor
TTP: time to peak
VSD: voltage-sensing domain
