# Supplementary material for: Voltage sensor gating charge interactions bimodally regulate voltage-dependence and kinetics of calcium channel activation
Source: J Gen Physiol. Author manuscript; Available in PMC 2025 Sep 1. (PMC7618026; doi:10.1085/jgp.202513769)
Supplement: Supplementary Materials [file EMS207767-suppement-Supplementary_Materials.pdf]

$$G = G_{max}/(1 + \exp\left(-\frac{V - V_{1/2}}{K}\right)). \quad (2)$$

The mean ± SEM for the calcium currents (I_{Ca}) sample traces was calculated by selecting the sweep at V_{max} for each recording constituting the dataset.

The time constant of activation ($\tau_{Activation}$) was evaluated be fitting the rising phase of the currents at V_{max} with a single exponential function.

Statistics

Statistical analysis and curve fitting was performed using SigmaPlot (version 12.0.0.182, Grafiti LLC). Figures were prepared in GraphPad Prism (version 10.2.3, GraphPad Software LLC) and Affinity Designer and Photo (version 2.5.3, Serif (Europe) Ltd.). All data are represented as mean ± SEM or mean ± SD. Figures presenting current traces the point by point calculated mean ± SEM. When comparing two different data sets, the statistical fit parameters were obtained by using a Student's t test. In case of non-Gaussian distribution of the data set, tested with a Shapiro-Wilk test, we performed a Mann-Whitney test. If the variances of the data set showed a significant difference, tested with a F-test, a Welch t-test was performed. To compare multiple data sets we performed one-way ANOVA combined with Dunnett's multiple comparison post hoc test with significance criteria. In case of non-Gaussian distribution of the data set, tested with a Shapiro-Wilk test, we performed a Kurskal-Wallis test with Dunn's multiple comparison post hoc test. If the variances of the data set showed a significant difference, tested with a Brown-Forsythe test, a Welch ANOVA test with Dunnett's T3 multiple comparison post hoc test was performed. The significance criteria are as follows *p < 0.05, **p < 0.01, ***p < 0.001, and ****p < 0.0001. The exact p-values for all statistical tests are shown in Tables 1 and 2.

Online Supplemental Material

Figs. S1 and S2 show the expression and localization in skeletal muscle triads of wildtype and mutated constructs of the embryonic $Ca_V1.1e$ splice variant in dysgenic myotubes, plus quantitative image analysis of those constructs, showing a reduced current density in the electrophysiological recordings.

Fig. S3 shows the gating properties of $Ca_V1.1e$ with mutated putative interaction partner N123 in combination with individual mutations in the CTC. Table S1 shows the parameters of double-immunofluorescence labeling of WT and gating charge mutants of $Ca_V1.1e$.

Supplementary Figure 1. Expression and localization in skeletal muscle triads of wildtype and gating charge mutant constructs of the embryonic Ca$_V$1.1e splice variant in dysgenic myotubes.

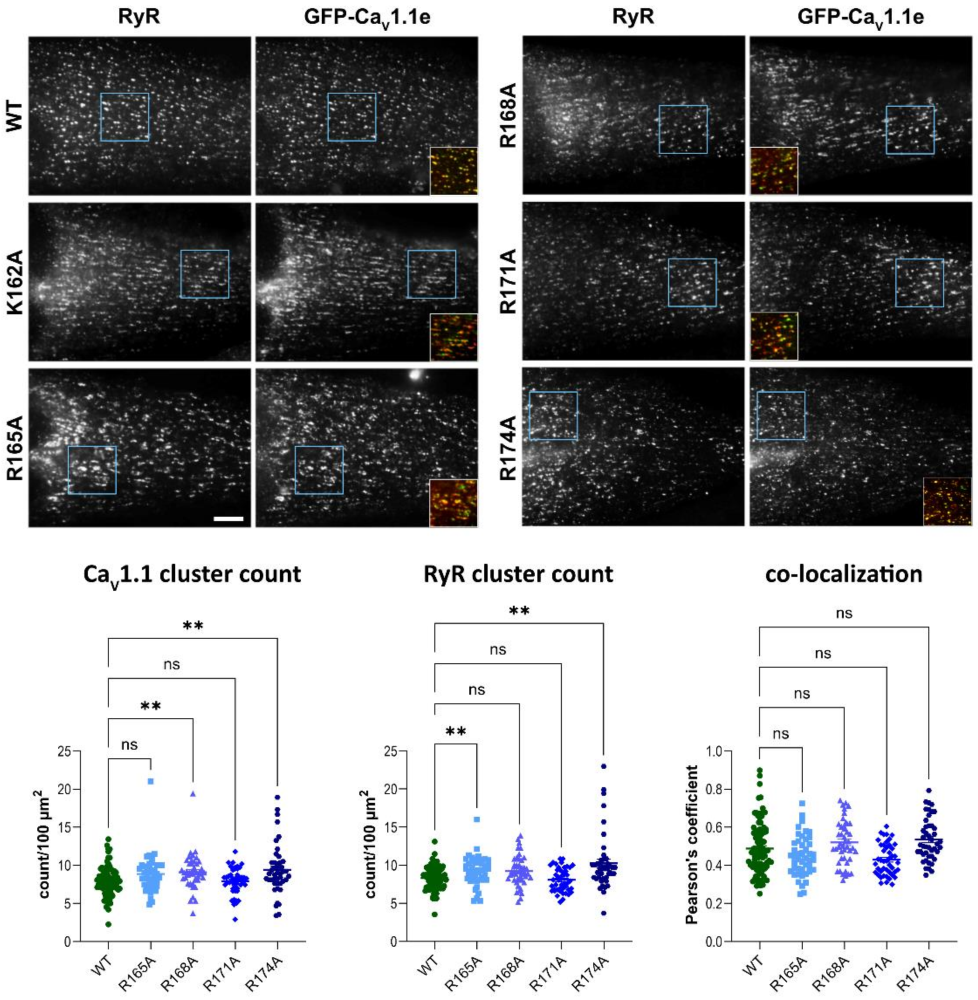

Double-immunofluorescence labeling of the type 1 ryanodine receptor (RyR1) and Ca$_V$1.1 (anti-GFP) in dysgenic (Ca$_V$1.1 -/-) myotubes reconstituted with the embryonal GFP-Ca$_V$1.1e splice variant or mutations thereof. **Top**: Representative myotubes of at least two separate experiments for each condition; scale bars, 10 µm. Co-clustering with RyR1 demonstrates the regular incorporation of the Ca$_V$1.1e constructs in skeletal muscle triads. **Bottom**: The cluster densities and co-localization (Pearson´s coefficient) were compared between wildtype Ca$_V$1.1e and mutant constructs (asterisks) to examine whether the decreased current densities observed in these mutants (Fig. 2M, N, U, V) were caused by reduced expression or triad targeting; which was not the case. Mean ± SEM; mutants and wt controls compared by one-way ANOVA multiple comparison, $* \triangleq p < 0.05$, $** \triangleq p < 0.01$, $*** \triangleq p < 0.001$. Exact p-values for all constructs are provided in SI Table 1.

SI Table 1 Parameters of double-immunofluorescence labeling of wildtype and gating charge mutants of Ca$_V$1.1e

Constructs	N[1]	n[1]	Ca$_V$1.1 cluster count/100 µm²	P-value	Δ	RyR cluster count/100 µm²	P-value	Δ	Co-localization (Pearson‘s coefficient)	P-value	Δ
Ca$_V$1.1e	6	90	7.97 ± 2.31	0.0747	0.89	8.33 ± 2.15	0.0088	0.96	0.49 ± 0.13	0.6491	0.040
Ca$_V$1.1e_ R165A	3	45	8.86 ± 2.56			9.29 ± 2.40			0.45 ± 0.12		
Ca$_V$1.1e_ R168A	3	45	9.10 ± 2.58	0.0028	1.13	9.24 ± 2.39	0.0503	0.91	0.52 ± 0.13	0.2405	0.032
Ca$_V$1.1e_ R171A	3	45	7.90 ± 2.34	>0.9999	0.067	8.08 ± 2.09	>0.9999	0.25	0.43 ± 0.11	0.0731	0.058
Ca$_V$1.1e_ R174A	3	45	9.17 ± 2.74	0.0069	1.44	10.07 ± 2.60	0.0013	1.93	0.53 ± 0.14	0.0511	0.047

[1]) N, number of experimental repeats (cell passages and transfections); n, number of recording

Supplementary Figure 2. Expression and localization in skeletal muscle triads of wildtype and countercharge mutant constructs of the embryonic $Ca_V1.1e$ splice variant in dysgenic myotubes.

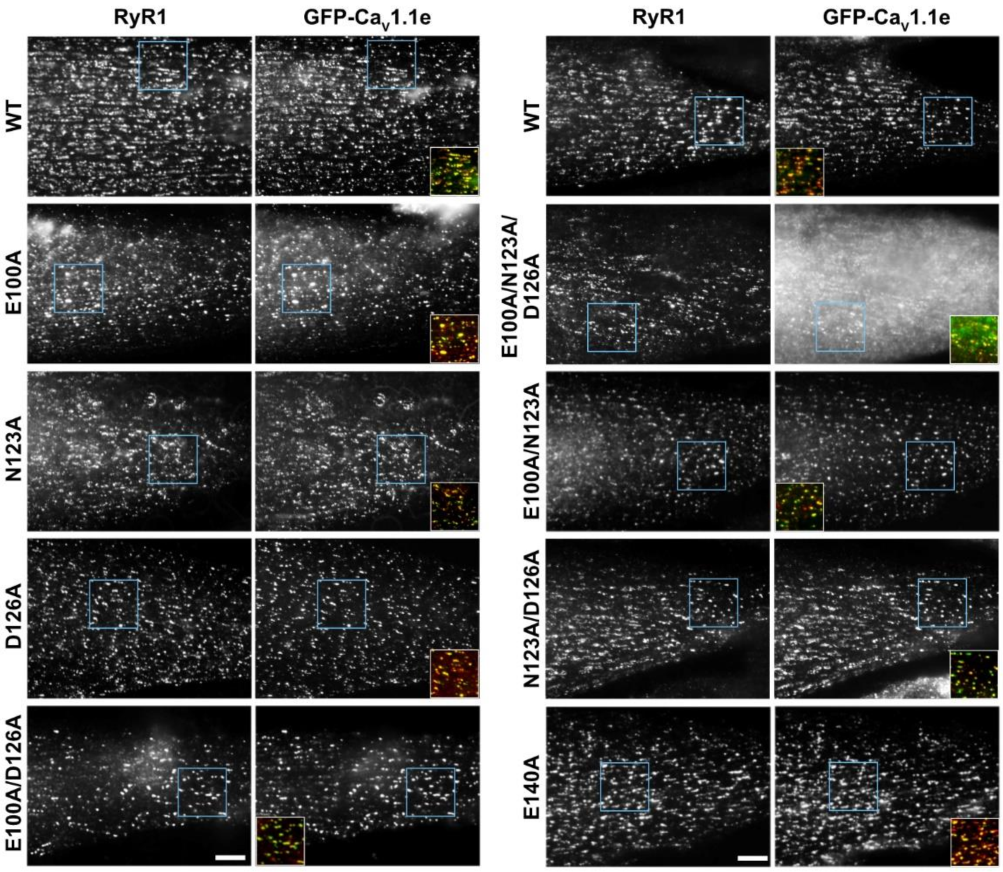

Double-immunofluorescence labeling of the ryanodine receptor (RyR1) and $Ca_V1.1$ (anti-GFP) in dysgenic ($Ca_V1.1$ -/-) myotubes reconstituted with the embryonal GFP-$Ca_V1.1e$ splice variant or mutations thereof. Representative myotubes of at least two separate experiments for each condition; scale bars, 10 µm. Co-clustering with RyR1 demonstrates the regular incorporation of the $Ca_V1.1e$ constructs in skeletal muscle triads. Only one construct used in this study, $Ca_V1.1e$_E100A/N123A/D126A, showed mostly diffuse membrane expression and a paucity of $Ca_V1.1$/RyR1 co-clusters. This, however, was not reflected by decreased current densities (Fig. 5F; Table 2).

Supplementary Figure 3. Gating properties of $Ca_V1.1e$ with mutated putative interaction partner N123 in combination with individual mutations in the CTC.

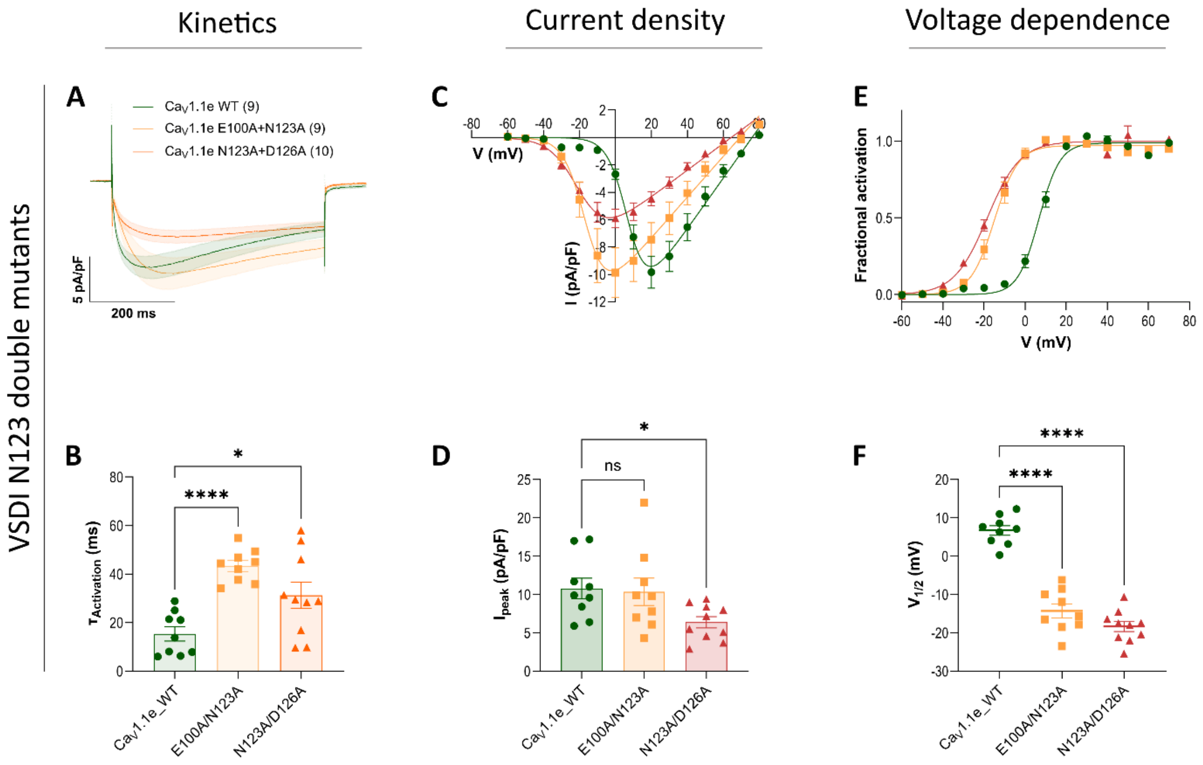

Displayed are mean calcium currents, the I/V- and G/V-curves (±SEM) and scatter plots time constants of activation ($\tau_{Activation}$), maximal current density (I_{peak}) and the voltage-dependence of activation ($V_{½}$) of whole-cell patch-clamp recordings in dysgenic ($Ca_V1.1$-null) myotubes reconstituted with wildtype (WT) and mutant $Ca_V1.1e$. **A-F:** The combined mutation E100A/N123A and N123A/D126A left-shifted the voltage-dependence and slowed the kinetics of current activation to a similar degree as mutations of the two CTC countercharges alone (cf. Fig. 4). Current densities were slightly reduced in case of the N123A/D126A mutant. For each mutation, recordings from 9-10 (n) myotubes of at least three separate experiments (passages and transfections) were compared with parallel-recorded controls. The data points are represented as mean ± SEM; mutants and wt controls compared by one-way ANOVA with Dunnett's multiple comparison, $* \triangleq p < 0.05$, $** \triangleq p < 0.01$, $*** \triangleq p < 0.001$, $**** \triangleq p < 0.0001$. Exact p-values for all constructs are provided in Table 2.
